# Indirect Effects of Universal Infant Rotavirus Vaccination: A Narrative Systematic Review

**DOI:** 10.3390/vaccines13050503

**Published:** 2025-05-09

**Authors:** Darren Suryawijaya Ong, Matthew Harris, John D. Hart, Fiona M. Russell

**Affiliations:** 1 Asia-Pacific Health, Infection, Immunity and Global Health, Murdoch Children’s Research Institute, Parkville, VIC 3052, Australiajohn.hart@mcri.edu.au (J.D.H.); fmruss@unimelb.edu.au (F.M.R.); 2 Department of Paediatrics, The University of Melbourne, Parkville, VIC 3052, Australia

**Keywords:** rotavirus, vaccine, immunisation, indirect effect, herd immunity

## Abstract

**Background/Objective:** Rotavirus is a major cause of acute gastroenteritis (AGE) in children <5 years. While rotavirus vaccines are effective in reducing AGE, limited data on their indirect effects exist. The aim of our narrative systematic review was to summarise the indirect effects of rotavirus vaccines on unvaccinated children and adults (PROSPERO: CRD42023418015). **Methods:** Peer-reviewed articles and conference abstracts were searched through Medline, Embase and PubMed on 8 December 2024. Observational studies of national/regional vaccine introduction were included. We included five outcomes: rotavirus–AGE inpatient admissions, rotavirus–AGE outpatient attendances, all-cause AGE inpatient admissions, all-cause AGE outpatient attendances, and stool rotavirus positivity. Outcome measures reported as percent reduction or individual incidence rates for the pre- and post-introduction periods were transformed to incidence rate ratios (IRRs). Median IRRs and interquartile ranges (IQRs) were calculated for each outcome by age group (<5, 5–19, and >18 years). **Results:** From an initial 757 articles, 44 studies including 9,327,974 participants were included. In unvaccinated children <5 years, there were reductions in rotavirus–AGE admissions (median IRR: 0.62, IQR: 0.40–0.82), rotavirus–AGE outpatient attendances (0.74, 0.16–0.98), all-cause AGE admissions (0.70, 0.56–0.86), and stool rotavirus positivity (0.42, 0.31–0.57), but not all-cause AGE outpatient attendances (0.92, 0.78–1.17). Few studies reported these outcomes for children and adolescents aged 5–19 years and adults >18 years. Indirect effects appeared to be greater in higher income and lower under-five mortality settings. **Conclusions:** Understanding these indirect benefits is crucial for evaluating the broader impact and cost-effectiveness of rotavirus immunisation programs.

## 1. Introduction

Rotavirus (RV) is the leading cause of morbidity and mortality due to acute gastroenteritis (AGE) in children less than five years old. In 2016, there were nearly 260 million episodes of AGE and 130,000 RV deaths in this age group, globally [1]. RV disease is not limited to children who are age-eligible for vaccination but also affects infants too young to be vaccinated and older adults [2,3]. There is a greater burden of RV-specific AGE (RV-AGE) earlier in life in infants from low- and middle-income countries compared to high-income countries [3]. The Global Burden of Disease 2019 Study found that in high and middle-income countries, RV death rates in people aged >70 years were higher than for children <5 years [4].

RV vaccines are effective in protecting vaccinated children (direct effects) against RV-AGE infections, hospitalisations and deaths [5,6]. As of 2023, 122 countries have introduced RV vaccines into their national immunisation programs (NIPs) [7]. There are four safe and effective RV vaccines that are prequalified by the WHO, namely RotaTeq™ (RV5), Rotarix™ (RV1), Rotavac™ and RotaSiil™, which are given from six weeks of age [8]. However, vaccine efficacy is lower in low-income and high-mortality countries, but the reasons for this are not well understood [9]. Additionally, due to the considerable burden of disease in the vaccine age-ineligible population, there is a need to determine the protection conferred to unvaccinated individuals as a result of others being vaccinated (indirect effects) from the infant immunisation program.

The indirect effects of RV vaccines are less well described, especially in low-income settings [10]. A meta-analysis of interventional studies in 2018 estimated that RV vaccine indirect effectiveness against RV-AGE hospitalisation in children <5 years was approximately 48% [11]. A systematic review of the indirect effects of RV vaccines in 2024 found a 40% decrease in RV-AGE hospitalisations, a 25% decrease in RV positivity, and a 42% decrease in the number of RV-positive tests following vaccine introduction [12].

While these studies found evidence of indirect protection against RV-AGE hospitalisations in unvaccinated individuals, the impact of RV vaccines against all-cause AGE hospitalisations and RV or all-cause AGE outpatient attendances in unvaccinated individuals is not known. These data are important for cost-effectiveness analyses, especially in countries with limited health resources, as including indirect effects may increase cost-effectiveness estimates, which directly inform immunisation policy [13]. The aim of our narrative systematic review was to summarise the indirect effects of universal infant RV vaccination on unvaccinated children and adults, focusing on inpatient admissions and outpatient attendances for RV-specific and all-cause AGE.

## 2. Materials and Methods

The narrative systematic review protocol was registered on PROSPERO (CRD42023418015) and is reported according to the PRISMA 2020 guidelines for systematic reviews (Supplementary S1) [14].

### 2.1. Search Strategy and Selection Criteria

We systematically searched Medline, Embase and PubMed on 8 December 2024 for peer-reviewed journal articles and conference abstracts. We searched terms related to “rotavirus”, “vaccine/immunisation” and “unvaccinated/indirect/herd” (Supplementary S2). Searches were limited to English language articles with no date limit. Reference lists of included studies and relevant systematic reviews were reviewed to identify additional articles of interest. We included studies reporting the indirect effects of universal infant RV vaccination on unvaccinated individuals. Unvaccinated individuals included both RV vaccine age-ineligible people, as well as age-eligible but unvaccinated people, as defined by the individual studies. We included all observational studies, and excluded randomised controlled trials, modelling studies, case studies, case series, qualitative studies, systematic reviews, opinion-based pieces, and animal studies. Only studies that were conducted in settings where there had been a national or regional introduction of the RV vaccine were included.

Outcomes of interest in unvaccinated individuals were (1) inpatient admissions for laboratory-confirmed RV–AGE, (2) outpatient attendances for laboratory-confirmed RV–AGE, (3) inpatient admissions for all-cause AGE, (4) outpatient attendances for all-cause AGE, and (5) laboratory-confirmed RV in stool samples. All-cause AGE refers to any AGE whereby the aetiology was not confirmed, as opposed to RV-AGE with laboratory-confirmed RV infection.

The search results were saved and uploaded to the Covidence software (https://www.covidence.org/) for screening. The first reviewer (D.S.O.) removed all duplicate articles and screened all titles, abstracts and full texts according to the inclusion and exclusion criteria. To reduce bias, a random selection of 10% titles and abstracts, and 100% of full-text articles, were independently screened by a second reviewer (M.H.). The reviewers documented the reason for the exclusion of any studies at the full-text screening stage. Disagreements at any stage of the review were resolved between both reviewers by consensus.

### 2.2. Data Extraction and Analysis

Data were extracted by the first reviewer using a data extraction table on Microsoft Excel. The data variables included article information (publication reference), study characteristics (country, study design, study period, study population, age group), RV vaccine information (date of universal vaccine introduction, formulation, coverage), case numbers, outcomes of interest, outcome measures (percent, percent change, prevalence, incidence rate, incidence rate ratio [IRR], incidence rate reduction, risk ratio, indirect vaccine effectiveness), and results. Outcome measures, 95% confidence intervals (CI) and *p* values were extracted, where available. Studies were assigned their World Bank country classification by their income level (low, lower-middle, upper-middle, and high) and UNICEF country under-five mortality rate [15,16].

When studies reported outcomes for separate and combined age groups, we only extracted data from the most specific age groups available (e.g., for one and two years separately, rather than 1–2 years combined). When studies measured outcomes over multiple consecutive years following vaccine introduction, we extracted the outcomes for each individual year (years post-introduction). When outcomes were measured over several consecutive years and some age groups subsequently included children who were eligible for vaccination during infancy, we only extracted data for age groups that were definitively unvaccinated. For example, if outcomes were reported for children aged 1–2 years, at zero years post-introduction, no children were vaccine-eligible during infancy; at one year post-introduction, one year olds were vaccine-eligible; and at two years post-introduction, 1–2 year olds were vaccine-eligible. For study periods starting in the first quarter of the calendar year, the calendar year of vaccine introduction was considered “Year 0”, followed by subsequent years. If the vaccine was introduced in the fourth quarter, the following calendar year was considered “Year 0”. For study periods beginning in the second, third or fourth quarters, when the vaccine was introduced in the preceding quarter, the current calendar year was considered “Year 0”.

For studies reporting the percent reduction or individual incidence rates for the pre- and post-introduction periods, we transformed these values to IRR using the following formulae:

(1) $$IRR=1-\frac{Percent\;reduction}{100}\;,$$

(2) $$IRR=\frac{Incidence\;rate\;in\;the\;post\;introduction\;period}{Incidence\;rate\;in\;the\;pre\;introduction\;period}\;.$$

As there was considerable heterogeneity between studies, including the reported outcome measures, age groups, and timing of measurement (years post-introduction), we did not perform a meta-analysis but instead summarised the distribution of each outcome using the medians and interquartile ranges (IQR) of the IRR by age (unvaccinated children <5 years, children and adolescents 5–19 years, and adults >18 years old). For unvaccinated children <5 years, we further visualised these distributions by years post-introduction (0, 1–2, 3–5, >5 years), the country income level (low, lower-middle, upper-middle, high), national under-five mortality rate (<10, >10–25, >25–50, >50–75, >75–100, >100 deaths per 1000 live births) [17], vaccine formulation (RV1, RV5, RV1 and RV5), national last dose rotavirus vaccine coverage (where available; <50%, 50–79%, 80–89%, ≥90%) [18], and risk of bias (low, some, high, very high). We performed a sensitivity analysis to exclude data from zero years post-introduction. We performed statistical analysis and generated graphs on Stata 18.0.

A risk of bias assessment was conducted by the first reviewer using the Risk Of Bias in Non-randomised Studies-of Exposures (ROBINS-E) tool [19]. A low risk of bias classification required all seven domains to be classified as low risk. Some concerns of bias classification had at least one domain classified as having some concerns, but all other domains remained low risk. A high risk or very high risk of bias classification had at least one domain with the respective risk. Studies that did not account for any confounding factors were classified as having a very high risk of bias.

## 3. Results

**Figure 1:**
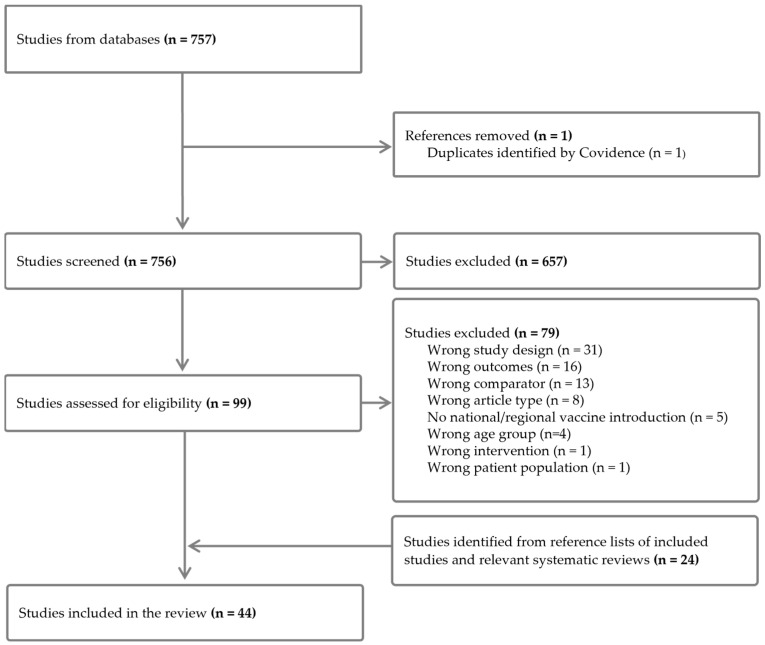
Flow diagram of included studies. “Wrong comparator” refers to studies that made the wrong comparison for the purposes of this review, such as calculating a ratio between unvaccinated versus vaccinated participants, rather than comparing the pre- versus post-vaccine introduction periods.

The literature search identified 756 studies after removing duplicates (Figure 1). An additional 24 studies were identified from reference lists of included papers and relevant systematic reviews. A total of 44 studies were eligible for inclusion, with a total of 9,327,974 participants. The reasons for excluding studies after full-text screening are listed in Supplementary S3.

Most included studies were peer-reviewed articles (n = 43, 98%) (Table 1). The study designs included hospital-based surveillance/observational studies (n = 25, 57%), register-based studies (n = 5, 11%), population-based surveillance/observational studies (n = 4, 9%), laboratory-based studies (n = 4, 9%), a clinical audit (n = 1, 2%), and a population and hospital-based observational study (n = 1, 2%). Four studies (9%) did not specify their design. Almost half of all studies were conducted in high-income countries (n = 20, 45%), while others were conducted in upper-middle-income (n = 6, 14%), lower-middle-income (n = 12, 27%), and low-income (n = 6, 14%) countries. Studies were conducted in countries with varying levels of under-five mortality, ranging from countries with ≤10 (n = 21, 48%), >10–25 (n = 4, 9%), >25–50 (n = 12, 27%), and >50–75 (n = 7, 16%) deaths per 1000 live births. Most studies were conducted in countries which introduced RV1 Rotarix™ (n = 29, 66%), followed by a combination of RV1 Rotarix™ and RV5 RotaTeq™ (n = 10, 23%), and RV5 RotaTeq™ (n = 5, 11%). The national last dose rotavirus vaccine coverage ranged from 0% to 98%. Most studies involved unvaccinated children <5 years (n = 32, 73%), followed by children <18 years (n = 7, 16%), all people ≥5 years (n = 2, 5%), adults ≥18 years (n = 1, 2%), older adults ≥65 years (n = 1, 2%), and all age groups (n = 1, 2%). No studies specifically focused on infants <6 weeks. The study characteristics and results are summarised in Supplementary S4.

The quality of studies varied considerably (Supplementary S5). Studies were classified as having some concerns (n = 35, 80%), having a high risk (n = 8, 18%), or having a very high risk of bias (n = 1, 2%). No studies were classified as having a low risk of bias. For most studies, the authors did not account for changes in access to healthcare or testing rates over time, which is an important confounding variable for the outcomes of interest. Some studies also did not account for RV seasonality. The lack of adjustment for these confounding variables mostly contributed to the overall risk of bias.

### 3.1. Inpatient Admissions for Laboratory-Confirmed RV-AGE

**Figure 2:**
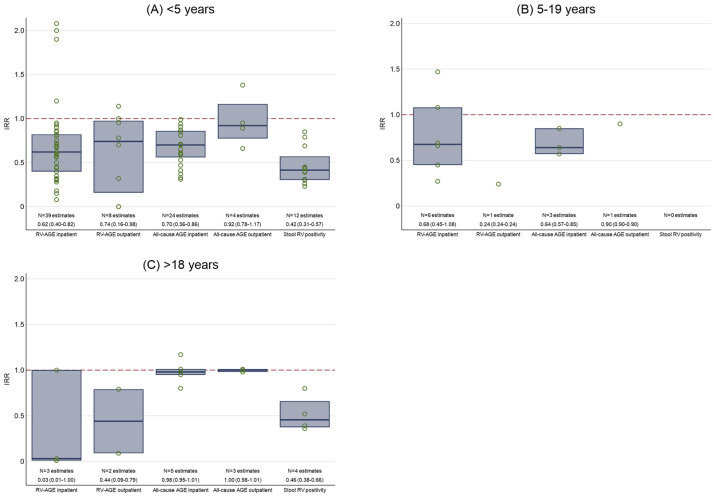
Distribution of the indirect effects of universal infant rotavirus vaccine introduction in (**A**) unvaccinated children <5 years, (**B**) children and adolescents 5–19 years, and (**C**) adults >18 years. Outcome measures shown are incidence rate ratios (IRR) of laboratory-confirmed rotavirus-specific acute gastroenteritis (RV-AGE) inpatient admissions, laboratory-confirmed RV-AGE outpatient attendances, all-cause AGE inpatient admissions, all-cause AGE outpatient attendances, and laboratory-confirmed RV positivity in stool samples, between the post- vs. pre-vaccine introduction periods. The boxes indicate the medians (centre line) and interquartile ranges (top and bottom lines) for each category. The number of individual estimates and median (interquartile range) for each outcome measure are shown. Total number of studies reporting the outcome: RV-AGE inpatient admissions (n = 20), RV-AGE outpatient attendances (n = 5), all-cause AGE inpatient admissions (n = 11), all-cause AGE outpatient attendances (n = 3), stool RV positivity (n = 9).

Twenty-three studies reported outcomes related to inpatient admissions for laboratory-confirmed RV-AGE [20,21,22,23,24,25,26,27,28,29,30,31,32,33,34,35,36,37,38,39,40,41,42,43,44]. The median reduction in RV-AGE inpatient admissions was 38% (median IRR: 0.62, IQR: 0.40–0.82, n = 39 estimates) for unvaccinated children <5 years (Figure 2A), 32% (median IRR: 0.68, IQR: 0.45–1.08, n = 6 estimates) for children and adolescents aged 5–19 years (Figure 2B), and 97% (median IRR: 0.03, IQR: 0.01–1.00, n = 3 estimates) for adults >18 years (Figure 2C). In children <5 years, the reduction in RV-AGE inpatient admissions was greater with a higher country income level, lower under-five mortality, and vaccine coverage <90%, but we did not observe any trends with years post-introduction, vaccine formulation, or risk of bias (Supplementary S6 Figure S1). Three studies reported indirect vaccine effectiveness by comparing unvaccinated children in the post-introduction period to children in the pre-introduction period. The indirect vaccine effectiveness amongst unvaccinated children was 66.5% (95% CI: 65.9–67.2) at 5–8 years post-introduction in Finnish children aged 0.5–2 years [26], 48.0% (95% CI: 42.8–52.6) at 0–8 years post-introduction in German children aged 0–5 years [27], and 14% at one year and 82% at four years post-introduction in children aged 0–20 months in the United States [37].

### 3.2. Outpatient Attendances for Laboratory-Confirmed RV-AGE

Five studies reported outcomes related to outpatient attendances for laboratory-confirmed RV-AGE [6,24,26,45,46]. The median reduction in RV-AGE outpatient attendances was 26% (median IRR: 0.74, IQR: 0.16–0.98, n = 8 estimates) for unvaccinated children <5 years (Figure 2A), 76% (median IRR: 0.24, IQR: 0.24–0.24, n = 1 estimate) for children and adolescents aged 5–19 years (Figure 2B), and 56% (median IRR: 0.44, IQR: 0.09–0.79, n = 2 estimates) for adults >18 years (Figure 2C). In children <5 years, the reduction in RV-AGE outpatient attendances was greater with a higher country income level, although the IQRs overlap, lower under-five mortality, and higher study risk of bias, although there was a limited number of estimates for some categories (Supplementary S6 Figure S2). We did not observe any trends with years post-introduction, vaccine formulation, or vaccine coverage (Supplementary S6 Figure S2). One study reported indirect vaccine effectiveness amongst unvaccinated children, which was 81.6% (95% CI: 81.6–81.7) at 5–8 years post-introduction in Finnish children aged 0.5–2 years [26].

### 3.3. Inpatient Admissions for All-Cause AGE

Twelve studies reported outcomes related to inpatient admissions for all-cause AGE [24,26,28,32,37,39,41,47,48,49,50,51]. The median reduction in all-cause AGE inpatient admissions was 30% (median IRR: 0.70, IQR: 0.56–0.86, n = 24 estimates) for unvaccinated children <5 years (Figure 2A), 36% (median IRR: 0.64, IQR: 0.57–0.85, n = 3 estimates) for children and adolescents aged 5–19 years (Figure 2B), and 2% (median IRR: 0.98, IQR: 0.95–1.01, n = 5 estimates) for adults >18 years (Figure 2C). In children <5 years, the reduction in all-cause inpatient admissions was greater with more years post-introduction, a higher country income level, lower under-five mortality, RV5 than RV1, and a higher study risk of bias, although there was a limited number of estimates for some categories (Supplementary S6 Figure S3). We did not observe any trends with vaccine coverage. Two studies reported indirect vaccine effectiveness amongst unvaccinated children, which was 56.4% (95% CI: 55.6–57.3) at 5–8 years post-introduction in Finnish children aged 0.5–2 years [26], and −8% at one year and 45% at four years post-introduction in children aged 0–20 months in the United States [37].

### 3.4. Outpatient Attendances for All-Cause AGE

Three studies reported outcomes related to outpatient attendances for all-cause AGE [24,26,41]. The median reduction in all-cause AGE outpatient attendances was 8% (median IRR: 0.92, IQR: 0.78–1.17, n = 4 estimates) for unvaccinated children <5 years (Figure 2A), 10% (median IRR: 0.90, IQR: 0.90–0.90, n = 1 estimate) for children and adolescents aged 5–19 years (Figure 2B), and 0% (median IRR: 1.00, IQR: 0.98–1.01, n = 3 estimates) for adults >18 years (Figure 2C). In children <5 years, the trends associated with years post-introduction, country income level, under-five mortality, vaccine formulation, vaccine coverage, and study risk of bias could not be assessed due to the low number of studies reporting this outcome (Supplementary S6 Figure S4). One study reported indirect vaccine effectiveness amongst unvaccinated children, which was 10.6% (95% CI: 9.6–11.6) at 5–8 years post-introduction in Finnish children aged 0.5–2 years [26].

### 3.5. Laboratory-Confirmed RV in Stool Samples

All nine studies reported a decline in the detection of laboratory-confirmed RV in stool samples [42,50,52,53,54,55,56,57,58]. The median reduction in stool RV positivity was 58% (median IRR: 0.42, IQR: 0.31–0.57, n = 12 estimates) for unvaccinated children <5 years (Figure 2A), and 54% (median IRR: 0.46, IQR: 0.38–0.66, n = 4 estimates) for adults >18 years (Figure 2C). No studies reported this outcome for children and adolescents aged 5–19 years (Figure 2B). In children <5 years, we did not observe any trends associated with years post-introduction, country income level, under-five mortality, vaccine formulation, vaccine coverage, or study risk of bias (Supplementary S6 Figure S5).

### 3.6. Mixed Outcomes

Five studies reported a combination of outcome measures [52,59,60,61,62]. In Ecuador, there was a 17.4% (95% CI: −16.9 to 41.6) decrease in community all-cause AGE in children and adults >5 years within five years of vaccine introduction [52]. In Finland, there was a reduction in combined RV-AGE emergency department outpatient attendances and inpatient admissions (72–94%) in children <16 years within five years of vaccine introduction [59,60]. In Israel, there was a reduction in inpatient admissions and outpatient attendances for RV-AGE and all-cause AGE in both Jewish and Bedouin children aged 1–4 years at 0–1 years post-introduction [61]. In Malawi, unvaccinated infants experienced a decrease in inpatient admissions and outpatient attendances for RV-AGE and all-cause AGE (10–47%) in the six years following vaccine introduction, while children aged 1–4 years experienced an increase (120–700%) [62].

### 3.7. Sensitivity Analysis

When we excluded data points that only reported data from zero years post-introduction, the median IRRs and IQRs of all outcome measures remained similar to the findings from the primary analyses (Supplementary S7 Figure S6).

## 4. Discussion

This narrative systematic review found evidence of the indirect effects of infant RV vaccination in unvaccinated children and adults, specifically inpatient admissions and outpatient attendances for RV-AGE, inpatient admissions for all-cause AGE, and RV positivity in stool samples, but not outpatient attendances for all-cause AGE. In children <5 years, this reduction appeared to be greater in higher-income countries and settings with lower under-five mortality levels. However, other than all-cause inpatient admissions, there were no clear differences in outcomes based on the time since vaccine introduction or vaccine formulation.

Most of the studies we found showed indirect effects of varying degrees in children <5 years. This age group had the greatest burden of RV disease prior to vaccine introduction, so the protection of unvaccinated children in this age group is an important feature of RV vaccines. A cluster-randomised trial in Bangladesh found no evidence of indirect protection for unvaccinated children (two weeks to two years of age) in villages where rotavirus vaccine was introduced [63]. All studies in our review showed indirect effects of varying degrees in older children. Although the overall RV disease burden has decreased since vaccines were introduced, the remaining disease burden has shifted to older age groups as infants and young children are directly protected through vaccination. This may suggest the limited indirect protection of RV vaccines and that herd immunity alone cannot be relied on to control RV disease in unvaccinated groups with a high disease burden. The trends for each outcome in adults generally followed similar patterns to children <5 years. However, this observation is less certain as there were a limited number of studies involving adults. Nevertheless, other studies have observed a greater reduction in inpatient admissions for RV-AGE (Canada) and all-cause AGE (Fiji) in younger adults aged 20–54 compared to older adults ≥55 years [24,47]. This could be due to younger adults having close contact with young children, who are the primary drivers of RV infection [64]. The lesser magnitude of indirect effects in older age groups in Fiji may indicate that RV infection in these populations are not solely driven by infants [64].

We did not find studies that looked at indirect effects specifically in infants too young to be vaccinated. Prior to the introduction of RV vaccination, 69% of RV-positive hospital admissions in children <5 years were in the first year of life, with 3% by ten weeks, 8% by 15 weeks, and 27% by 26 weeks [65]. While the burden of AGE is primarily in infants aged 2–6 months, passive protection provided by maternal antibodies wane over the first six weeks of life [3,66]. Considerations for a neonatal dose are required if there is a lack of indirect effects in this age-ineligible group, so it is important for future studies assessing indirect effects to include <6 weeks as a standalone age group.

Our findings are similar to the meta-analysis by Rosettie and colleagues which found lower indirect vaccine effectiveness against RV-AGE hospitalisation in children <5 years in low- and middle-income countries compared to high-income countries [11]. Similarly, Chavers and colleagues found a greater reduction in RV-AGE hospitalisations in settings of lower under-five mortality, although similar to our analysis, IQRs overlapped between categories [12]. Studies have demonstrated lower vaccine efficacy and effectiveness in low-income countries with high under-five mortality rates [67]. The reasons for this are not well understood but is likely multifactorial, including differences in RV epidemiology, enteric coinfections, malnutrition, maternal antibodies, and coadministration of other vaccines [67,68].

There did not seem to be a clear difference in outcomes by time since vaccine introduction. Some studies in our review observed a fluctuation of indirect effects within the same age group over time. The percent change in total inpatient admissions for RV-AGE in children aged two years in three United States counties increased in the first year post-vaccine introduction, then decreased in the second year, before increasing again in the third year [38]. The authors suggested that RV vaccine introduction may have provided one year of indirect protection against RV disease, but protection did not persist. RV vaccine efficacy wanes over time and more rapidly in high-mortality settings, but how this waning may impact any indirect effects is not known [69].

Indirect effects did not seem to vary by vaccine formulation. Similar results by vaccine formulation were reported in a meta-analysis of the direct effectiveness of RV vaccines, which showed comparable vaccine effectiveness of the RV1 Rotarix™ and RV5 RotaTeq™ vaccines in countries of low, medium, and high child mortality [5]. Pooled estimates of indirect effects against RV-AGE in children <5 years also found no difference in protection between both vaccine formulations [70].

Some modelling studies have sought to estimate the indirect effects of RV vaccines. However, our review focused on compiling empirical evidence and excluded this study design. A modelling study including 112 low- and middle-income countries estimated that indirect effects against rotavirus deaths were greater in countries with higher under-five mortality [71]. Similar to our review, Rosettie and Chavers both found the opposite trend [11,12], although all three reviews focused on rotavirus morbidity rather than mortality. Importantly, our review only included studies that assessed universal vaccine introduction, excluding private-market introductions which could have underestimated any effects due to low vaccine coverage. However, vaccine coverage did not seem to impact indirect effects, except for greater reductions in RV-AGE when coverage was <90%. Our observation needs to be interpreted with caution as we had a low number of estimates in the ≥90% coverage category. Conversely, the modelling study of 112 countries found that indirect effects against rotavirus deaths were greater with increased vaccine coverage [71].

Similarly, RV vaccine research programs may need to consider trials for vaccinating older adults if indirect effects are not substantial in this age group. RV disease burden in adults ≥60 years is not well characterised, but it is estimated that between 0–62% of AGE patients in this age group are RV positive [2]. We found two studies from Canada and Fiji that did not find indirect effects in the ≥55 year age group [24,47]. However, an Irish study observed a decrease in stool sample RV positivity in older adults following national infant RV vaccine introduction [54]. Context-specific considerations to vaccinate older adults against RV are important and depend on disease burden in this age group.

Our review had several limitations. Firstly, we could not conduct a meta-analysis due to substantial heterogeneity in the type of outcome measure, age group, and time since vaccine introduction. However, our review highlights the presence of indirect effects across multiple outcomes throughout the age spectrum and across the number of years since vaccine introduction. Secondly, none of the studies were classified as having low risk of bias as they were all observational and prone to confounding. While most studies stratified for age and some for RV seasonality, most studies did not adjust for changes in access to healthcare during the study period. This is an important confounder as these changes impact infectious disease reporting [72,73]. Increased access to healthcare throughout the study period, including the provision of oral rehydration solution, may also reduce the severity of RV disease [74]. Due to the lack of adjustment, some of the observations in this review could have been attributable to increased access to healthcare rather than vaccination. Thirdly, some studies presented findings from surveillance programs which could have had changes in testing, reporting, and admission criteria during the study period that were not adjusted for. Fourthly, testing bias could have been introduced as RV testing is not routinely performed for AGE patients [75]. Some studies analysed data from surveillance programs, while others opportunistically tested a convenient group of patients or laboratory samples. Fifthly, we only included English language publications and conference abstracts due to logistical limitations, which may have missed data from non-English speaking regions. Finally, unlike other reviews, we only extracted results for age groups and timepoints that did not include vaccinated or vaccine-eligible individuals, which limited the number of estimates and included studies. However, this ensured that any potential direct effects due to the inclusion of vaccinated individuals were minimised.

## 5. Conclusions

This narrative systematic review enabled the compilation of evidence on the indirect effects of universal infant RV vaccination on unvaccinated children and adults. We found evidence of indirect effects against inpatient admissions and outpatient attendances for RV-AGE, inpatient admissions for all-cause AGE, and detection of RV in stool samples. Indirect effects appeared to be greater in higher-income countries and settings with lower under-five mortality rates. The significant variability in methodologies across studies highlights the importance of standardising measures of indirect effects to enable comparison and synthesis. Policy decisions to introduce and improve RV immunisation programs should include indirect effects as an important consideration.

## Figures and Tables

**Table 1:** Characteristics of included studies.

Study Characteristics	N = 44
**Article type**	
Peer-reviewed	43 (98%)
Conference abstract	1 (2%)
**Study design**	
Hospital-based surveillance or observational study	25 (57%)
Register-based study	5 (11%)
Population-based surveillance or observational study	4 (9%)
Laboratory-based study	4 (9%)
Observational study (unspecified)	4 (9%)
Clinical audit	1 (2%)
Population and hospital-based observational study	1 (2%)
**Country income level**	
High	20 (45%)
Upper-middle	6 (14%)
Lower-middle	12 (27%)
Low	6 (14%)
**National under-five mortality level** (deaths per 1000 live births)	
≤10	21 (48%)
>10–25	4 (9%)
>25–50	12 (27%)
>50–75	7 (16%)
**Vaccine formulation**	
RV1 Rotarix™	29 (66%)
RV5 RotaTeq™	5 (11%)
RV1/RV5 combination	10 (23%)
**Age group**	
Children <5 years	32 (73%)
Children <18 years	7 (16%)
All people ≥5 years	2 (5%)
All people ≥18 years	1 (2%)
Older adults ≥65 years	1 (2%)
All age groups	1 (2%)
**Outcome measure ^1^**	
Inpatient admissions for laboratory-confirmed RV-AGE	23 (52%)
Outpatient attendances for laboratory-confirmed RV-AGE	5 (11%)
Inpatient admissions for all-cause AGE	12 (27%)
Outpatient attendances for all-cause AGE	3 (7%)
Laboratory-confirmed RV in stool samples	9 (20%)
Indirect vaccine effectiveness against the above outcomes	3 (7%)
Mixed outcomes	5 (11%)

^1^ Total count exceeds N = 44 and total percentage exceeds 100% due to individual studies reporting multiple outcome measures. Note: Total percentages may not add up to 100% due to rounding. RV (rotavirus), AGE (acute gastroenteritis).

## Data Availability

The original contributions presented in this study are included in the article/Supplementary Material. Further inquiries can be directed to the corresponding author.

## Supplementary Materials

The following supporting information can be downloaded at: https://www.mdpi.com/article/10.3390/vaccines13050503/s1, S1: PRISMA 2020 guidelines checklist; S2: Literature search strategy for PubMed, Medline and Embase; S3: List of studies excluded after full-text screening and their reasons; S4: Study characteristics and outcomes of indirect effects of universal infant rotavirus vaccination in unvaccinated children and adults; S5: Risk of bias assessment of included studies using the ROBINS-E tool; S6: Subgroup analyses of distribution of the indirect effects of universal infant rotavirus vaccine introduction; S7: Sensitivity analysis of distribution of the indirect effects of universal infant rotavirus vaccine introduction, excluding data points that only reported data from zero years post-introduction.

## Abbreviations

The following abbreviations are used in this manuscript:

AGE	Acute gastroenteritis
CI	Confidence interval
IQR	Interquartile range
IRR	Incidence rate ratio
NIP	National immunisation program
ROBINS-E	Risk Of Bias in Non-randomised Studies-of Exposures
RV	Rotavirus
RV1	Rotarix™ vaccine
RV5	RotaTeq™ vaccine
RV-AGE	Rotavirus-specific acute gastroenteritis
