# Supplementary material for: Indirect Effects of Universal Infant Rotavirus Vaccination: A Narrative Systematic Review
Source: Vaccines (Basel). 2025 May 9;13(5):503. doi: 10.3390/vaccines13050503 (PMC12116122; doi:10.3390/vaccines13050503)
Supplement: Supplementary file 1 [file vaccines-13-00503-s001.zip › vaccines-3581697-supplementary.pdf]

**Supplementary File**

**Indirect effects of universal infant rotavirus vaccination: a narrative systematic review**

Supplementary S1. PRISMA 2020 guidelines checklist …………………………………………………………………… 2

Supplementary S2. Literature search strategy for PubMed, Medline and Embase ……………………………………… 5

Supplementary S3. List of studies excluded after full-text screening and their reasons ……………………………… 7

Supplementary S4. Study characteristics and outcomes of indirect effects of universal infant rotavirus vaccination in unvaccinated children and adults ……………………………………………………………………………… 9

Supplementary S5. Risk of bias assessment of included studies using the ROBINS-E tool …………………………… 23

Supplementary S6. Subgroup analyses of distribution of the indirect effects of universal infant rotavirus vaccine introduction ……………………………………………………………………………………………… 26

Supplementary S7. Sensitivity analysis of distribution of the indirect effects of universal infant rotavirus vaccine introduction, excluding data points that only reported data from zero years post-introduction ……………………… 31

References ……………………………………………………………………………………………………… 32

**Supplementary S1. PRISMA 2020 guidelines checklist.**

| Section and Topic | Item # | Checklist item | Location where item is reported (page) |
|---|---|---|---|
| **TITLE** | | | |
| Title | 1 | Identify the report as a systematic review. | 1 |
| **ABSTRACT** | | | |
| Abstract | 2 | See the PRISMA 2020 for Abstracts checklist. | 1 |
| **INTRODUCTION** | | | |
| Rationale | 3 | Describe the rationale for the review in the context of existing knowledge. | 1-2 |
| Objectives | 4 | Provide an explicit statement of the objective(s) or question(s) the review addresses. | 2 |
| **METHODS** | | | |
| Eligibility criteria | 5 | Specify the inclusion and exclusion criteria for the review and how studies were grouped for the syntheses. | 2-4 |
| Information sources | 6 | Specify all databases, registers, websites, organisations, reference lists and other sources searched or consulted to identify studies. Specify the date when each source was last searched or consulted. | 2 |
| Search strategy | 7 | Present the full search strategies for all databases, registers and websites, including any filters and limits used. | 2 |
| Selection process | 8 | Specify the methods used to decide whether a study met the inclusion criteria of the review, including how many reviewers screened each record and each report retrieved, whether they worked independently, and if applicable, details of automation tools used in the process. | 3 |
| Data collection process | 9 | Specify the methods used to collect data from reports, including how many reviewers collected data from each report, whether they worked independently, any processes for obtaining or confirming data from study investigators, and if applicable, details of automation tools used in the process. | 3 |
| Data items | 10a | List and define all outcomes for which data were sought. Specify whether all results that were compatible with each outcome domain in each study were sought (e.g. for all measures, time points, analyses), and if not, the methods used to decide which results to collect. | 3 |
| | 10b | List and define all other variables for which data were sought (e.g. participant and intervention characteristics, funding sources). Describe any assumptions made about any missing or unclear information. | 3 |
| Study risk of bias assessment | 11 | Specify the methods used to assess risk of bias in the included studies, including details of the tool(s) used, how many reviewers assessed each study and whether they worked independently, and if applicable, details of automation tools used in the process. | 4 |
| Effect measures | 12 | Specify for each outcome the effect measure(s) (e.g. risk ratio, mean difference) used in the synthesis or presentation of results. | 3-4 |
| Synthesis methods | 13a | Describe the processes used to decide which studies were eligible for each synthesis (e.g. tabulating the study intervention characteristics and comparing against the planned groups for each synthesis (item #5)). | 4 |
| | 13b | Describe any methods required to prepare the data for presentation or synthesis, such as handling of missing summary statistics, or data conversions. | 3 |

| Section and Topic | Item # | Checklist item | Location where item is reported (page) |
|---|---|---|---|
| | 13c | Describe any methods used to tabulate or visually display results of individual studies and syntheses. | 4 |
| | 13d | Describe any methods used to synthesize results and provide a rationale for the choice(s). If meta-analysis was performed, describe the model(s), method(s) to identify the presence and extent of statistical heterogeneity, and software package(s) used. | 4 |
| | 13e | Describe any methods used to explore possible causes of heterogeneity among study results (e.g. subgroup analysis, meta-regression). | 4 |
| | 13f | Describe any sensitivity analyses conducted to assess robustness of the synthesized results. | 4 |
| Reporting bias assessment | 14 | Describe any methods used to assess risk of bias due to missing results in a synthesis (arising from reporting biases). | NA |
| Certainty assessment | 15 | Describe any methods used to assess certainty (or confidence) in the body of evidence for an outcome. | NA |
| **RESULTS** | | | |
| Study selection | 16a | Describe the results of the search and selection process, from the number of records identified in the search to the number of studies included in the review, ideally using a flow diagram. | 4 |
| | 16b | Cite studies that might appear to meet the inclusion criteria, but which were excluded, and explain why they were excluded. | 4 |
| Study characteristics | 17 | Cite each included study and present its characteristics. | 5-6 |
| Risk of bias in studies | 18 | Present assessments of risk of bias for each included study. | 6 |
| Results of individual studies | 19 | For all outcomes, present, for each study: (a) summary statistics for each group (where appropriate) and (b) an effect estimate and its precision (e.g. confidence/credible interval), ideally using structured tables or plots. | 6-8 |
| Results of syntheses | 20a | For each synthesis, briefly summarise the characteristics and risk of bias among contributing studies. | 6-8 |
| | 20b | Present results of all statistical syntheses conducted. If meta-analysis was done, present for each the summary estimate and its precision (e.g. confidence/credible interval) and measures of statistical heterogeneity. If comparing groups, describe the direction of the effect. | 6-8 |
| | 20c | Present results of all investigations of possible causes of heterogeneity among study results. | NA |
| | 20d | Present results of all sensitivity analyses conducted to assess the robustness of the synthesized results. | 8 |
| Reporting biases | 21 | Present assessments of risk of bias due to missing results (arising from reporting biases) for each synthesis assessed. | NA |
| Certainty of evidence | 22 | Present assessments of certainty (or confidence) in the body of evidence for each outcome assessed. | NA |
| **DISCUSSION** | | | |
| Discussion | 23a | Provide a general interpretation of the results in the context of other evidence. | 8-10 |

| Section and Topic | Item # | Checklist item | Location where item is reported (page) |
|---|---|---|---|
| | 23b | Discuss any limitations of the evidence included in the review. | 10 |
| | 23c | Discuss any limitations of the review processes used. | 10 |
| | 23d | Discuss implications of the results for practice, policy, and future research. | 10 |
| **OTHER INFORMATION** | | | |
| Registration and protocol | 24a | Provide registration information for the review, including register name and registration number, or state that the review was not registered. | 2 |
| | 24b | Indicate where the review protocol can be accessed, or state that a protocol was not prepared. | 2 |
| | 24c | Describe and explain any amendments to information provided at registration or in the protocol. | NA |
| Support | 25 | Describe sources of financial or non-financial support for the review, and the role of the funders or sponsors in the review. | 11 |
| Competing interests | 26 | Declare any competing interests of review authors. | 11 |
| Availability of data, code and other materials | 27 | Report which of the following are publicly available and where they can be found: template data collection forms; data extracted from included studies; data used for all analyses; analytic code; any other materials used in the review. | 11 |

**Supplementary S2. Literature search strategy for PubMed, Medline and Embase.**

(A) PubMed

1 title/abstract
"rotavirus"
2 title/abstract
"vaccin*" OR "immuniz*" OR "immunis*"
3 #1 AND #2
4 title/abstract
"rotavirus-vaccine*" OR "rotarix" OR "rotateq" OR "rv3" OR "rv3-bb" OR "rotashield" OR "rrv-tv"
5 #3 OR #4
6 title/abstract
"unvaccinated" OR "indirect" OR "herd" OR "adaptive" OR "unprotected"
7 all fields
NOTNLM OR publisher[sb] OR inprocess[sb] OR pubmednotmedline[sb] OR indatareview[sb] OR pubstatusaheadofprint
8 #5 AND #6 AND #7

(B) Ovid Medline

1 Rotavirus/
2 Rotavirus Infections/
3 rotavirus.tw,kf.
4 1 or 2 or 3
5 exp Immunization/
6 (vaccin* or immuniz* or immunis*).tw,kf.
7 5 or 6
8 4 and 7
9 Rotavirus Vaccines/
10 (rotavirus-vaccine* or rotarix or rotateq or rv3 or rv3-bb or rotashield or rrv-tv).tw,kf.
11 8 or 9 or 10
12 (unvaccinated or indirect).tw,kf.
13 Immunity, Herd/
14 (herd or adaptive or unprotected).tw,kf.
15 12 or 13 or 14
16 11 and 15
17 limit 16 to (case reports or comment or editorial or guideline or letter or practice guideline)
18 16 not 17

(C) Embase

1 rotavirus/ or human rotavirus/ or exp rotavirus a/ or rotavirus b/ or rotavirus c/
2 Rotavirus infection/
3 rotavirus.tw,kf,dq.
4 1 or 2 or 3
5 exp immunization/
6 (vaccin* or immuniz* or immunis*).tw,kf,dq.
7 5 or 6
8 4 and 7
9 Rotavirus vaccine/
10 (rotavirus-vaccine* or rotarix or rotateq or rv3 or rv3-bb or rotashield or rrv-tv).tw,kf,dq.
11 8 or 9 or 10
12 (unvaccinated or indirect).tw,kf,dq.
13 herd immunity/
14 (herd or adaptive or unprotected).tw,kf,dq.
15 12 or 13 or 14
16 11 and 15
17 case report/

18 limit 16 to (editorial or letter)
19 16 not (17 or 18)

**Supplementary S3. List of studies excluded after full-text screening and their reasons.**

| Study | DOI | Reason for exclusion |
|---|---|---|
| Adlhoch 2013 | https://dx.doi.org/10.1097/INF.0b013e3182720b71 | Wrong outcome |
| Aliabadi 2017 | https://dx.doi.org/10.1093/ofid/ofx163.763 | Wrong study design |
| Armstrong 2016 | https://dx.doi.org/10.1016/j.vaccine.2016.08.004 | Wrong study design |
| Atchison 2016 | https://dx.doi.org/10.1093/infdis/jiv398 | Wrong study design |
| Aziz 2021 | https://dx.doi.org/10.1016/j.vaccine.2021.08.048 | Wrong study design |
| Baker 2019 | https://dx.doi.org/10.1093/cid/ciy580 | Wrong study design |
| Bawa 2015 | https://dx.doi.org/10.1093/cid/civ264 | Wrong study design |
| Becheanu 2017 | https://dx.doi.org/10.1007/s00431-017-2979-8 | Wrong comparator |
| Begue 2010 | https://dx.doi.org/10.1542/peds.2009-2069 | Wrong comparator |
| Benedicto-Matambo 2022 | https://dx.doi.org/10.3390/vaccines10030418 | Wrong article type |
| Bennett 2016 | https://dx.doi.org/10.1016/j.vaccine.2016.07.001 | Wrong article type |
| Burns 2020 | https://dx.doi.org/10.1093/eurpub/ckz238 | Wrong study design |
| Buttery 2011 | https://dx.doi.org/10.1097/INF.0b013e3181fefdee | Wrong study design |
| Charoenwat 2022 | https://dx.doi.org/10.1097/MPG.0000000000003446 | Wrong age group |
| Cho 2018 | https://dx.doi.org/10.1016/j.jval.2018.07.475 | No national, state or regional vaccine introduction |
| Clark 2009 | https://dx.doi.org/10.1128/CVI.00382-08 | Wrong outcome |
| Clarke 2011 | https://dx.doi.org/10.1016/j.vaccine.2011.04.109 | Wrong study design |
| Cortes 2011 | https://dx.doi.org/10.1056/NEJMoa1000446 | Wrong study design |
| Cortese 2015 | https://dx.doi.org/10.1093/infdis/jiu503 | Wrong comparator |
| Costa 2016 | https://dx.doi.org/10.1097/INF.0000000000001143 | Wrong study design |
| Costantino 2018 | https://dx.doi.org/10.1080/21645515.2018.1471306 | Wrong age group |
| Crealey 2019 | https://dx.doi.org/10.1136/archdischild-2019-epa.249 | Wrong age group |
| Dey 2012 | https://dx.doi.org/10.5694/mja12.10062 | Wrong study design |
| Escolano 2020 | https://dx.doi.org/10.2807/1560-7917.Es.2020.25.33.1900538 | Wrong study design |
| Ezzine 2021 | https://dx.doi.org/10.26719/2021.27.6.553 | Wrong outcome |
| Fathima 2021 | https://dx.doi.org/10.2188/jea.JE20200066 | Wrong study design |
| Field 2010 | https://dx.doi.org/10.1542/peds.2010-0443 | Wrong study design |
| Forrest 2015 | https://dx.doi.org/10.1016/j.jcv.2015.07.172 | Wrong study design |
| Gosselin 2016 | https://dx.doi.org/10.1080/21645515.2016.1189038 | Wrong outcome |
| Gower 2022 | https://dx.doi.org/10.1093/cid/ciab460 | Wrong study design |
| Gurgel 2014 | https://dx.doi.org/10.1371/journal.pone.0110217 | Wrong outcome |
| Hamdan 2018 | https://dx.doi.org/10.1093/jpids/piy091 | Wrong outcome |
| Hegazi 2017 | https://dx.doi.org/10.1097/MD.0000000000006574 | Wrong comparator |
| Hemming-Harlo 2017 | https://dx.doi.org/10.1093/jpids/piw061 | Wrong comparator |
| Inns 2016 | https://dx.doi.org/10.1080/21645515.2015.1108501 | Wrong study design |
| Jain 2016 | https://dx.doi.org/10.1007/s13312-016-0893-1 | No national, state or regional vaccine introduction |
| John 2014 | https://dx.doi.org/10.1016/j.vaccine.2014.03.004 | No national, state or regional vaccine introduction |
| Justino 2016 | https://dx.doi.org/10.1093/tropej/fmv098 | Wrong outcome |
| Kraay 2018 | DOI not available | Wrong intervention/exposure |
| Krishnarajah 2017 | https://dx.doi.org/10.1016/j.vaccine.2017.06.034 | Wrong comparator |
| Lambert 2009 | https://dx.doi.org/10.5694/j.1326-5377.2009.tb02727.x | Wrong study design |
| Lopman 2011 | https://dx.doi.org/10.1093/infdis/jir492 | Wrong study design |
| Lopman 2012 | https://dx.doi.org/10.1016/j.coviro.2012.05.002 | Wrong article type |

| Study | DOI | Reason for exclusion |
|---|---|---|
| Lopman 2013 | https://dx.doi.org/10.1371/journal.pone.0067763 | Wrong comparator |
| Macartney 2011 | https://dx.doi.org/10.1111/j.1440-1754.2010.01953.x | Wrong comparator |
| Markkula 2017 | https://dx.doi.org/10.1080/23744235.2016.1275773 | Wrong outcome |
| Marlow 2015 | https://dx.doi.org/10.2807/1560-7917.Es.2015.20.48.30077 | Wrong outcome |
| Marquis 2020 | https://dx.doi.org/10.1097/INF.0000000000002656 | Wrong study design |
| Mast 2015 | https://dx.doi.org/10.1097/INF.0000000000000702 | Wrong outcome |
| Mrozek-Budzyn 2012 | DOI not available | Wrong outcome |
| Muhsen 2010 | https://dx.doi.org/10.1016/j.vaccine.2010.10.010 | No national, state or regional vaccine introduction |
| Muhsen 2017 | https://dx.doi.org/10.1080/21645515.2017.1297908 | Wrong article type |
| Mwenda 2018 | https://dx.doi.org/10.1016/j.vaccine.2018.06.026 | Wrong article type |
| Ngabo 2016 | https://dx.doi.org/10.1016/S2214-109X(15)00270-3 | Wrong age group |
| Orozco 2009 | https://dx.doi.org/10.1086/605053 | Wrong study design |
| Panozzo 2013 | https://dx.doi.org/10.1002/pds.3512 | Wrong study design |
| Patel 2009 | https://dx.doi.org/10.1001/jama.2009.756 | Wrong comparator |
| Paulke-Korinek 2011 | https://dx.doi.org/10.1016/j.vaccine.2011.01.104 | Wrong study design |
| Pindyck 2018 | https://dx.doi.org/10.1080/14760584.2018.1489724 | Wrong article type |
| Quintanar-Solares 2011 | https://dx.doi.org/10.1097/INF.0b013e3181fefb32 | Wrong study design |
| Reyes 2017 | https://dx.doi.org/10.1016/j.vaccine.2016.11.056 | Wrong study design |
| Rha 2014 | https://dx.doi.org/10.1586/14760584.2014.877846 | Wrong article type |
| Richardson 2010 | https://dx.doi.org/10.1056/NEJMoa0905211 | Wrong study design |
| Sabbe 2016 | https://dx.doi.org/10.2807/1560-7917.Es.2016.21.27.30273 | Wrong study design |
| Simwaka 2021 | https://dx.doi.org/10.1371/journal.pone.0246025 | Wrong patient population |
| Sohn 2014 | https://dx.doi.org/10.14776/kjpid.2014.21.3.174 | Wrong comparator |
| Standaert 2016 | https://dx.doi.org/10.1007/s40121-015-0099-1 | Wrong outcome |
| Standaert 2020 | https://dx.doi.org/10.1007/s40121-020-00345-y | Wrong outcome |
| Tate 2009 | https://dx.doi.org/10.1542/peds.2008-1200 | No national, state or regional vaccine introduction |
| Tate 2009 | https://dx.doi.org/10.1542/peds.2008-3528 | Wrong study design |
| Tate 2011 | https://dx.doi.org/10.1097/INF.0b013e3181fefdc0 | Wrong article type |
| Tate 2016 | https://dx.doi.org/10.1093/cid/civ1016 | Wrong comparator |
| Thomas 2017 | https://dx.doi.org/10.1016/j.vaccine.2016.11.057 | Wrong study design |
| Verberk 2021 | https://dx.doi.org/10.1016/j.vaccine.2021.09.059 | Wrong outcome |
| Vesikari 2013 | https://dx.doi.org/10.1097/INF.0000000000000086 | Wrong comparator |
| Vieira 2011 | https://dx.doi.org/10.1590/s0074-02762011000300012 | Wrong outcome |
| Walker 2019 | https://dx.doi.org/10.1016/j.jvacx.2019.100005 | Wrong outcome |
| Yen 2011 | https://dx.doi.org/10.1097/INF.0b013e3181fefa05 | Wrong study design |
| Yoshikawa 2018 | https://dx.doi.org/10.1016/j.vaccine.2017.12.006 | Wrong comparator |

**Supplementary S4. Study characteristics and outcomes of indirect effects of universal infant rotavirus vaccination in unvaccinated children and adults.** For study periods starting in the first quarter of the calendar year, the calendar year of vaccine introduction was considered "Year 0". If vaccine was introduced in the fourth quarter, the following calendar year was considered "Year 0". For study periods beginning in the second, third or fourth quarters, when vaccine was introduced in the preceding quarter, the current calendar year was considered "Year 0". Case numbers (n/N) were extracted for the relevant age group, unless otherwise specified. For the post-vaccine period, case numbers were reported exclusively for unvaccinated individuals, where available. Results were included only for age groups and timepoints that did not include vaccinated or vaccine-eligible individuals. Where available, 95% confidence intervals (CI) and p values were extracted. RV (rotavirus), RV-AGE (rotavirus-specific acute gastroenteritis), y (year), m (month), NBW (normal birth weight), LBW (low birth weight), VLBW (very low birth weight).

| Reference | Country | Date of universal rotavirus vaccine introduction | Vaccine formulation and coverage | Study design | Study period | Study population | Age groups | Case numbers | Outcome and effect measure | Results (95% CI) |
|---|---|---|---|---|---|---|---|---|---|---|
| **Inpatient admissions for laboratory-confirmed RV-AGE** | | | | | | | | | | |
| 1 | Armenia | Nov 2012 | RV1 Rotarix<br><br><1y<br>Year 1: 16%<br>Year 2: 72%<br>Year 3: 77%<br><br>1-<2y<br>Year 1: 0%<br>Year 2: 25%<br>Year 3: 81%<br><br>2-<5y<br>Year 1: 0%<br>Year 2: 0%<br>Year 3: 8% | Hospital-based case-control | Pre-vaccine:<br>Jul 2010 - Jun 2012<br><br>Transition (Year 0):<br>Jul 2012 - Jun 2013<br><br>Post-vaccine (Years 1-2):<br>Jul 2013 - Jun 2015 | Children admitted to two hospitals with AGE<br><br>Case: Laboratory-confirmed RV positive<br><br>Control: Laboratory-confirmed RV negative | <5y | **Pre-vaccine**<br>1-<2y: 320/668 (48%)<br>2-<5y: 254/688 (37%)<br><br>**Unvaccinated post-vaccine**<br><br>**1-<2y:**<br>Year 0 = 221/817 (27%)<br><br>**2-<5y:**<br>Year 0 = 169/803 (21%)<br>Year 1 = 197/756 (26%) | Incidence rate ratio of RV-AGE inpatient admissions | **1y**<br>Year 0: 0.56 (0.47-0.67)<br><br>**2-4y**<br>Year 0: 0.57 (0.47-0.70)<br>Year 1: 0.71 (0.58-0.85) |
| 2 | Australia | Jul 2007 | RV1 Rotarix<br><br><1y (2 doses)<br>2010: 88% | Retrospective clinical audit | Pre-vaccine:<br>Jan 2004 - Dec 2006<br><br>Post-vaccine (Years 1-5):<br>Jan 2008 - Dec 2012 | Children admitted to a tertiary hospital with RV enteritis | <5y | **Pre- and post-vaccine combined**<br>289 (total in all age groups) | Percent reduction in RV-AGE inpatient admissions | **2y**<br>Year 0: 85%<br><br>**3y**<br>Year 0: 43% |
| 3 | Austria | Aug 2007 | RV1 Rotarix and RV5 RotaTeq<br><br>2010: 78%<br>2011: 84% | Hospital-based surveillance | Pre-vaccine:<br>2001-2005<br><br>Post-vaccine (Years 3-4):<br>Jan 2010 - Dec 2011 | Children admitted to 11 paediatric wards with laboratory-confirmed RV-AGE | <15y | **Pre-vaccine**<br>Not available<br><br>**Post-vaccine**<br>823 (total in all age groups) | Incidence rate of RV-AGE inpatient admissions per 100,000 (and percent change) | **Incidence rate**<br><br>**5-9y**<br>Pre-vaccine: 53 (ref)<br>Year 3: 35 (-34%)<br>Year 4: 78 (+48%)<br><br>**10-14y**<br>Pre-vaccine: 13 (ref)<br>Year 3: 9 (-32%)<br>Year 4: 14 (+6%)<br><br>**Incidence rate ratio**<br><br>**5-9y** |

| Reference | Country | Date of universal rotavirus vaccine introduction | Vaccine formulation and coverage | Study design | Study period | Study population | Age groups | Case numbers | Outcome and effect measure | Results (95% CI) |
|---|---|---|---|---|---|---|---|---|---|---|
| | | | | | | | | | | Year 3: 0.66*<br>Year 4: 1.47*<br><br>**10-14y**<br>Year 3: 0.69*<br>Year 4: 1.08* |
| [4] | Brazil | Mar 2006 | RV1 Rotarix<br><br>2006: 54%<br>2007: 78%<br>2008: 81% | Hospital-based surveillance | Pre-vaccine:<br>Jan 2004 - Dec 2005<br><br>Post-vaccine (Years 1-2):<br>Jan 2007 - Dec 2008 | Children admitted to a hospital with AGE | <5y | **Pre-vaccine**<br>2-4y: 34/97 (35%)<br><br>**Unvaccinated post-vaccine**<br>2-4y: 24/50 (48%) | Percent reduction in RV-AGE inpatient admissions | **2-4y**<br>Years 1-2: 29.4% |
| [5] | Canada | Aug 2011<br><br>RV5 and RV1 in private market from 2006/2007 | RV1 Rotarix and RV5 RotaTeq<br><br><1y^<br>2017: 79% | Retrospective population-based cohort | Pre-vaccine:<br>Aug 2005 – Jul 2011<br><br>Post-vaccine (Years 0-4):<br>Aug 2011 – Mar 2016 | Ontarians with a valid health card that utilised healthcare services for AGE | All ages | **Pre-vaccine and unvaccinated post-vaccine combined**<br>2779/183,170 (total in all age groups) | Incidence rate ratio of RV-AGE inpatient admissions | **5-19y**<br>Years 0-4: 0.27 (0.18-0.41)<br><br>**20-44y**<br>Years 0-4: 0.01 (0.00-0.40)<br><br>**45-64y**<br>Years 0-4: 0.03 (0.00-0.32)<br><br>**≥65y**<br>Years 0-4: 1.00 (0.23-4.38) |
| [6] | Estonia | Jul 2014 | RV5 RotaTeq (Jul 2014 - Oct 2015)<br><br>RV1 Rotarix (Oct 2015 onwards)<br><br>2015: 65.6%<br>2016: 86.8% | Hospital-based observational study | Pre-vaccine:<br>2007-2013<br><br>Post-vaccine (Years 1-2):<br>2015-2016 | Children admitted to seven hospitals with AGE | <18y | **Pre-vaccine**<br>7620/21,037 (total in all age groups)<br><br>**Post-vaccine**<br>567/2249 (total in all age groups) | Percent reduction in RV-AGE inpatient admissions | **5-9y**<br>Years 1-2: 55% (37-68) |
| [7] | Finland | 2009 | RV5 RotaTeq<br><br><1y^<br>2014: 92%<br>2015: 91%<br>2016: 84%<br>2017: 80% | Retrospective register-based study | Pre-vaccine:<br>2002-2005<br><br>Post-vaccine (Years 5-8):<br>2014-2017 | Inpatient and outpatient children due to AGE in a national healthcare register | 0.5-2y | **Pre-vaccine**<br>6m: 55,376 person-years<br>12m: 55,376 person-years<br>18m: 55,376 person-years<br><br>**Unvaccinated post-vaccine**<br>6m: 4304 person-years<br>12m: 4304 person-years<br>18m: 4304 person-years | Incidence of RV-AGE inpatient admissions per 1000 person-years<br><br>Indirect vaccine effectiveness against RV-AGE inpatient admissions | **Incidence** (Years 5-8)<br>6m: 9.03 (pre); 1.63 (post)<br>12m: 5.92 (pre); 2.09 (post)<br>18m: 4.48 (pre); 2.79 (post)<br><br>**Incidence rate ratio** (Years 5-8)<br>6m: 0.18*<br>12m: 0.35*<br>18m: 0.62*<br><br>**Indirect vaccine effectiveness**<br>66.5% (65.9-67.2) |
| [8] | Germany | Statewide:<br>Jan 2008<br><br>Nationwide:<br>2013 | RV1 Rotarix and RV5 RotaTeq<br><br>Overall: 47.6%<br>0-<1y: 62.7%<br>1-<2y: 55.1%<br>2-<3y: 47.5%<br>3-<4y: 39.1% | Descriptive observational study | Pre-vaccine:<br>Oct 2002 - Dec 2007<br><br>Post-vaccine (Years 0-8):<br>Jan 2008 - Sep 2016 | Children admitted to a hospital with AGE | <5y | **Pre-vaccine and unvaccinated post-vaccine combined**<br>0-<1y: 289<br>1-<2y: 260<br>2-<3y: 117<br>3-<4y: 46<br>4-<5y: 36 | Indirect vaccine effectiveness against RV-AGE inpatient admissions | Overall: 48% (42.8-52.6)<br><1y: 45%<br>1y: 35%<br>2y: 26%<br>3y: 29%<br>4y: 25% |

| Reference | Country | Date of universal rotavirus vaccine introduction | Vaccine formulation and coverage | Study design | Study period | Study population | Age groups | Case numbers | Outcome and effect measure | Results (95% CI) |
|---|---|---|---|---|---|---|---|---|---|---|
| | | | 4-<5y: 31.4% | | | | | | | |
| 9,10 | Ghana | Apr 2012 | RV1 Rotarix<br><br>2012-2016: 93% | Hospital-based surveillance | Pre-vaccine:<br>Jul 2009 - Jun 2012<br><br>Post-vaccine (Years 0-3):<br>Jul 2012 - Jun 2016 | Children admitted for diarrhoea in two tertiary hospitals | <5y | **Pre-vaccine**<br>1y: 340/711<br>2-4y: 93/274<br><br>**Unvaccinated post-vaccine**<br><br>**1y**<br>Year 0: 56/177<br><br>**2-4y**<br>Year 0: 15/48<br>Year 1: 14/52 | Percent reduction in RV-AGE inpatient admissions | **1y**<br>Year 0: 33% (p=0.002)<br><br>**2-4y**<br>Year 0: 7% (p=0.77)<br>Year 1: 20.1% (p=0.40) |
| 11 | Kenya | Jul 2014 | RV1 Rotarix<br><br>2 doses<br>2014: 82%<br>2016: 100% | Hospital-based surveillance | Pre-vaccine:<br>Jul 2009 – Jun 2014<br><br>Post-vaccine (Years 0-1):<br>Jul 2014 – Jun 2016 | Children hospitalised with severe AGE at a county hospital | <5y | **Pre-vaccine**<br>429/1546 (total for all age groups)<br><br>**Post-vaccine**<br>91/658 (total for all age groups) | Percent reduction in RV-AGE inpatient admissions | **1y**<br>Year 0: 14% (6-25)<br><br>**2-4y**<br>Year 0: 31% (17-47)<br>Year 1: 15% (6-27) |
| 12 | Moldova | Jul 2012 | RV1 Rotarix<br><br>1 dose<br>Year 1: 20%<br>Year 2: 40% | Hospital-based surveillance | Pre-vaccine:<br>Sep 2009 - Jul 2012<br><br>Post-vaccine (Years 0-2):<br>Aug 2012 – Jul 2014 | Children admitted to two paediatric hospitals with acute diarrhoea | <5y | **Pre-vaccine**<br>12-23m: 725/1524 (48%)<br>24-59m: 732/1390 (53%)<br><br>**Unvaccinated post-vaccine**<br><br>**12-23m:**<br>Year 0 = 111/327 (33%)<br><br>**24-59m:**<br>Year 0 = 116/346 (33%)<br>Year 1 = 84/349 (22%) | Incidence rate reduction of RV-AGE inpatient admissions | **1y**<br>Year 0: 25% (14-35)<br><br>**2-4y**<br>Year 0: 32% (22-41)<br>Year 1: 55% (30-72) |
| 13 | Tanzania | Jan 2013 | RV1 Rotarix<br><br><1y<br>2013: 18% (Apr), 65% (Oct)<br>2014: 85% (Apr), 83% (Oct) | Hospital-based surveillance | Pre-vaccine:<br>2010 - 2012<br><br>Post-vaccine (Years 0-2):<br>2013 - 2015 | Children admitted to a hospital with diarrhoea as the main reason for admission | <5y | **Pre-vaccine**<br>1y: 145<br>2-4y: 45<br><br>**Unvaccinated post-vaccine**<br><br>**1y:**<br>Year 0 = 105<br><br>**2-4y:**<br>Year 0 = 28<br>Year 1 = 54 | Percent reduction in RV-AGE inpatient admissions | **1y**<br>Year 0: 28%<br><br>**2-4y**<br>Year 0: 38%<br>Year 1: -20% |
| 14 | Tanzania | Jan 2013 | RV1 Rotarix<br><br><1y^<br>2014: 97%<br>2015: 95% | Hospital-based surveillance | Pre-vaccine:<br>2009 – 2012<br><br>Post-vaccine (Years 1-2):<br>2014 – 2015 | Children admitted due to diarrhoea in two regional hospitals | <5y | **Pre-vaccine**<br>Tanga: 25/65<br>Bugando: 2/8<br><br>**Unvaccinated post-vaccine**<br>Tanga: 1/3<br>Bugando: 3/20 | Percent reduction in RV-AGE inpatient admissions | **2-4y**<br>Year 1: Tanga 5%; Bugando 10%* |

| Reference | Country | Date of universal rotavirus vaccine introduction | Vaccine formulation and coverage | Study design | Study period | Study population | Age groups | Case numbers | Outcome and effect measure | Results (95% CI) |
|---|---|---|---|---|---|---|---|---|---|---|
| [15] | Thailand | Oct 2011 (one province only) | RV1 Rotarix<br><br>2 doses: 97% | Population-based cohort | Sep 2012 - Oct 2014 | Children being immunised at public immunisation clinics in two provinces (vaccine vs no vaccine introduction) | <5y | **Unvaccinated post-vaccine**<br>3y: 10/50 (20%)<br>4y: 10/28 (35.7%) | Percent change in RV-AGE inpatient admissions | 3y: -69%<br>4y: -40% |
| [16] | United Kingdom (Scotland) | Jul 2013 | RV1 Rotarix<br><br>2 doses<br>93-94% | Laboratory-based study | Pre-vaccine:<br>Jul 2012 - Jun 2013<br><br>Post-vaccine (Years 0-1):<br>Jul 2013 - Jun 2015 | Children with RV-positive stool samples identified from a regional laboratory record | <13y | **Pre-vaccine**<br>1y: 56<br>2y: 16<br>3-12y: 13<br><br>**Unvaccinated post-vaccine**<br><br>**1y**<br>Year 0: 17<br><br>**2y**<br>Year 0: 8<br>Year 1: 57<br><br>**3-12y**<br>Year 0: 2<br>Year 1: 3 | Percent reduction in RV-AGE inpatient admissions | **1y**<br>Year 0: 69.6% (47-83.5)<br><br>**2y**<br>Year 0: 50.0% (-23.8 to 81.5)<br>Year 1: 56.3% (-12.4 to 84.8)<br><br>**3-12y**<br>Year 0: 84.6% (32.0-98.3)<br>Year 1: 76.9% (16.0-95.8) |
| [17] | United Kingdom | Jul 2013 | RV1 Rotarix<br><br>Complete course 2015: 89% | Retrospective hospital-based observational study | Pre-vaccine:<br>Jul 2002 – Jun 2013<br><br>Post-vaccine (Years 0-1):<br>Jul 2013 – Jun 2015 | Children admitted with RV-AGE or RV-AGE developed after hospitalisation in primary, secondary and tertiary care facilities | <15y | **Pre-vaccine and unvaccinated post-vaccine combined**<br>1644 (total in all age groups) | Percent reduction in RV-AGE inpatient admissions | **2-4y:**<br>Years 0-1: 69% (38-86) (p=0.017) |
| [18] | United States | RV5: Feb 2006<br><br>RV1: Apr 2008 | RV5 RotaTeq and RV1 Rotarix<br><br>≥1 dose<br>2007: 51%<br>2008: 66%<br>2009: 80%<br>2010: 86% | Retrospective register-based study | Pre-vaccine:<br>2001 – 2005<br><br>Post-vaccine (Years 1-4):<br>2007 – 2010 | Children on a commercial health insurance database with at least one outpatient claim[#] | <20m | **Pre-vaccine**<br>722/277,899<br><br>**Unvaccinated post-vaccine**<br>Year 1: 60/64,929<br>Year 2: 91/91,051<br>Year 3: 74/61,218<br>Year 4: 13/41,946 | Indirect vaccine effectiveness against RV-AGE inpatient admissions | **<20m**<br>Year 1: 14% (-14 to 36)<br>Year 2: 44% (30-55)<br>Year 3: 40% (24-53)<br>Year 4: 82% (70-90) |
| [19] | United States | RV5: Feb 2006<br><br>RV1: Apr 2008 | RV5 RotaTeq and RV1 Rotarix<br><br>2006: 0%<br>2007: 10%<br>2008: 42%<br>2009: 67% | Population-based surveillance | Pre-vaccine:<br>2006<br><br>Post-vaccine (Years 1-3):<br>2007 - 2009 | Children admitted to three hospitals with AGE | <3y | **Pre-vaccine and unvaccinated post-vaccine combined**<br>2y: 65/130 (50%) | Percent change in RV-AGE inpatient admissions | **2y**<br>Year 1: 108.1%<br>Year 2: -91.9%<br>Year 3: 89.5% |
| [20] | United States | RV5: Feb 2006<br><br>RV1: Apr 2008 | RV5 RotaTeq and RV1 Rotarix<br><br><1y with ≥1 dose<br>2007-2008: NBW | Retrospective register-based study | Pre-vaccine:<br>Jul 2001 – Jun 2006<br><br>Post-vaccine (Years 1-8):<br>Jul 2007 – Jun 2015 | Children on a commercial health insurance database[#] | <5y | **Pre-vaccine and post-vaccine combined**<br>2,256,000 (total in all age groups) | Incidence rate reduction in RV-AGE inpatient admissions | **2-4y**<br>Year 1: NBW 68% (63-72)<br><br>Note: not calculated for LBW and VLBW |

| Reference | Country | Date of universal rotavirus vaccine introduction | Vaccine formulation and coverage | Study design | Study period | Study population | Age groups | Case numbers | Outcome and effect measure | Results (95% CI) |
|---|---|---|---|---|---|---|---|---|---|---|
| | | | 64%; LBW 62%; VLBW 47%<br>2008-2009: NBW 72%; LBW 69%; VLBW 52%<br>2009-2010: NBW 76%; LBW 73%; VLBW 58%<br>2010-2011: NBW 78%; LBW 76%; VLBW 63%<br>2011-2012: NBW 82%; LBW 78%; VLBW 64%<br>2012-2013: NBW 86%; LBW 81%; VLBW 66%<br>2013-2014: NBW 88%; LBW 83%; VLBW 67%<br>2014-2015: NBW: 89%; LBW 84%; VLBW 68% | | | | | | | |
| [21] | United States | RV5: Feb 2006<br><br>RV1: Apr 2008 | RV5 RotaTeq and RV1 Rotarix<br><br>2005-2006: 0.4%<br>2006-2007: ~25%<br>2008-2009: 54% | Retrospective register-based study | Pre-vaccine:<br>Jul 2003 - Jun 2006<br><br>Post-vaccine (Years 1-2):<br>Jul 2007 - Jun 2009 | Military dependents hospitalised with RV-AGE | <5y | **Pre-vaccine** (total in all age groups)<br>2003-2004: 589/563,301<br>2004-2005: 669/564,162<br>2005-2006: 800/556,721<br><br>**Post-vaccine** (total in all age groups)<br>2007-2008: 208/563,099<br>2008-2009: 321/586,719 | Percent reduction in RV-AGE inpatient admissions | **<1y**<br>Years 1-2: 60.3% (51.4-67.9)<br><br>**1y**<br>Years 1-2: 54.5% (46.4-61.5)<br><br>**2y**<br>Years 1-2: 40.5% (26.0-52.4)<br><br>**3y**<br>Years 1-2: 18.2% (-13.1 to 41.4) |
| [22] | United States | RV5: Feb 2006<br><br>RV1: Apr 2008 | RV5 RotaTeq and RV1 Rotarix<br><br><1y with ≥1 dose<br>2007: 64%<br>2008: 73%<br>2009: 76%<br>2010: 78% | Retrospective register-based study | Pre-vaccine:<br>Jul 2001 – Jun 2006<br><br>Post-vaccine (Years 1-4):<br>Jul 2007 – Jun 2011 | Children on a commercial health insurance database with diarrhoea-associated healthcare events# | <5y | **Pre-vaccine and post-vaccine combined**<br>406,000 (total in all age groups) | Incidence rate reduction in RV-AGE inpatient admissions | **2-4y**<br>Year 1: 72% (67-76) |
| [23] | United States | RV5: Feb 2006<br><br>RV1: Apr 2008 | RV5 RotaTeq and RV1 Rotarix<br><br><5y (≥1 dose)<br>2008: 32% | Laboratory-based study | Post-vaccine, pre-impact (Years 0-1):<br>Feb 2006 - May 2007<br><br>Post-vaccine impact (Years 2-4):<br>Feb 2008 - May 2010 | Adults with stool samples collected for bacterial stool culture, acting as a surrogate for clinically significant diarrhoea | ≥18y | **Pre-vaccine**<br>43/989 (4.35%)<br><br>**Unvaccinated post-vaccine**<br>57/2541 (2.24%) | Prevalence of RV-AGE inpatient admissions | **≥18y**<br>Years 0-1: 7.96/10,000<br>Years 2-4: 5.69/10,000 (p=0.28) |

| Reference | Country | Date of universal rotavirus vaccine introduction | Vaccine formulation and coverage | Study design | Study period | Study population | Age groups | Case numbers | Outcome and effect measure | Results (95% CI) |
|---|---|---|---|---|---|---|---|---|---|---|
| [24,25] | Zambia | Regional: Jan 2012<br><br>Nationwide: Nov 2013 | RV1 Rotarix<br><br>2 doses<br>2013: 39%<br>2014: 77%<br>2015: 88%<br>2016: 94% | Hospital-based surveillance | Pre-vaccine:<br>Jan 2009 - Dec 2011<br><br>Post-vaccine (Years 1-4):<br>Jan 2013 - Dec 2016 | Children admitted to a teaching hospital with AGE | <5y | **Pre-vaccine**<br>2-4y: ~15/144<br><br>**Unvaccinated post-vaccine**<br>2-4y: 10/58 | Percent reduction in RV-AGE inpatient admissions | **2-4y**<br>Year 1: -100% |
| **Outpatient attendances for laboratory-confirmed RV-AGE** | | | | | | | | | | |
| [5] | Canada | Aug 2011<br><br>RV5 and RV1 in private market from 2006/2007 | RV1 Rotarix and RV5 RotaTeq<br><br><1y^<br>2017: 79% | Retrospective population-based cohort | Pre-vaccine:<br>Aug 2005 – Jul 2011<br><br>Post-vaccine (Years 0-4):<br>Aug 2011 – Mar 2016 | Ontarians with a valid health card that utilised healthcare services for AGE | All ages | **Pre-vaccine and unvaccinated post-vaccine combined**<br>2779/183,170 (total in all age groups) | Incidence rate ratio of RV-AGE emergency department outpatient attendances | **5-19y**<br>Years 0-4: 0.24 (0.09-0.64)<br><br>**20-44y**<br>Years 0-4: 0.09 (0.02-0.47)<br><br>**45-64y**<br>Years 0-4: 0 (0-.)<br><br>**≥65y**<br>Years 0-4: 0.79 (0.06-10.34)<br><br>Note: Due to small numbers of events in the pre-program period, a rate ratio could not be calculated for 45-64y age group |
| [7] | Finland | 2009 | RV5 RotaTeq<br><br><1y^<br>2014: 92%<br>2015: 91%<br>2016: 84%<br>2017: 80% | Retrospective register-based study | Pre-vaccine:<br>2002-2005<br><br>Post-vaccine (Years 5-8):<br>2014-2017 | Inpatient and outpatient children due to AGE in a national healthcare register | 0.5-2y | **Pre-vaccine**<br>6m: 55,376 person-years<br>12m: 55,376 person-years<br>18m: 55,376 person-years<br><br>**Unvaccinated post-vaccine**<br>6m: 4304 person-years<br>12m: 4304 person-years<br>18m: 4304 person-years | Incidence of RV-AGE outpatient attendances per 1000 person-years<br><br>Indirect vaccine effectiveness against RV-AGE outpatient attendances | **Incidence** (Years 5-8)<br>6m: 0.65 (pre); 0.00 (post)<br>12m: 0.38 (pre); 0.00 (post)<br>18m: 0.23 (pre); 0.23 (post)<br><br>**Incidence rate ratio** (Years 5-8)<br>6m: 0*<br>12m: 0*<br>18m: 1*<br><br>**Indirect vaccine effectiveness**<br>81.6% (81.6-81.7) |
| [26] | Malawi | Oct 2012 | RV1 Rotarix<br><br>0 dose: 3.8%<br>1 dose: 5.3%<br>2 doses: 90.9% | Hospital-based surveillance | Pre-vaccine:<br>Jan - Jun 2012<br><br>Post-vaccine (Years 0-3):<br>Jan 2013 - Jun 2016 | Children presenting to a government hospital with AGE | <5y | **Pre-vaccine**<br>117/221 (52.9%)<br><br>**Unvaccinated post-vaccine**<br>65/184 (35.3%) | Relative risk of RV incidence in AGE outpatient attendances | **<1y**<br>Years 0-3: 0.70 (0.55-0.88; p=0.003)<br><br>**1-4y**<br>Years 0-3: 1.14 (0.79-1.63; p value not available) |
| [27] | United Kingdom | Jul 2013 | RV1 Rotarix<br><br>2 doses: 88% | Retrospective hospital-based surveillance | Pre-vaccine:<br>2012-2013<br><br>Post-vaccine (Year 1):<br>2014 | Children presenting to emergency at a hospital with AGE | <18y | **Pre-vaccine**<br>2012: 77<br>2013: 92<br><br>**Unvaccinated post-vaccine**<br>2014: 35 | Percent reduction in RV-AGE outpatient attendances | **1-4y**<br>Year 1: 67-70% |

| Reference | Country | Date of universal rotavirus vaccine introduction | Vaccine formulation and coverage | Study design | Study period | Study population | Age groups | Case numbers | Outcome and effect measure | Results (95% CI) |
|---|---|---|---|---|---|---|---|---|---|---|
| [28] | Zimbabwe | May 2014 | RV1 Rotarix<br><br>2 doses<br>2006: 89% | Hospital-based surveillance | Pre-vaccine:<br>Jan 2012 - Dec 2013<br><br>Post-vaccine (Years 1-2):<br>Jan 2015 - Dec 2016 | Children presenting to three hospitals with AGE | <5y | Not available | Percent reduction in RV-AGE outpatient attendances | **2-4y**<br>Year 1: 22% (p=0.19)<br>Year 2: 5% (p=0.52) |
| **Inpatient admissions for all-cause AGE** | | | | | | | | | | |
| [5] | Canada | Aug 2011<br><br>RV5 and RV1 in private market from 2006/2007 | RV1 Rotarix and RV5 RotaTeq<br><br><1y^<br>2017: 79% | Retrospective population-based cohort | Pre-vaccine:<br>Aug 2005 – Jul 2011<br><br>Post-vaccine (Years 0-4):<br>Aug 2011 – Mar 2016 | Ontarians with a valid health card that utilised healthcare services for AGE | All ages | **Pre-vaccine and unvaccinated post-vaccine combined**<br>2779/183,170 (total in all age groups) | Incidence rate ratio of all-cause AGE inpatient admissions | **5-19y**<br>Years 0-4: 0.85 (0.80-0.91)<br><br>**20-44y**<br>Years 0-4: 1.01 (0.98-1.05)<br><br>**45-64y**<br>Years 0-4: 0.95 (0.92-0.98)<br><br>**≥65y**<br>Years 0-4: 0.98 (0.94-1.01) |
| [29] | Fiji | Oct 2012 | RV1 Rotarix<br><br>2013: 85%<br>2014: 91%<br>2015: 94%<br>2016: 93%<br>2017: 98% | Retrospective observational study | Pre-vaccine:<br>Jan 2006 - Dec 2011<br><br>Post-vaccine (Years 1-5):<br>Jan 2014 - Dec 2018 | All admissions to tertiary hospitals nationwide | ≥5y | Mean annual cases<br><br>**Pre-vaccine**<br>5-9y: 85.6<br>10-19y: 79.4<br>20-54y: 194.8<br>≥55y: 78.8<br><br>**Unvaccinated post-vaccine**<br>5-9y: 57.0<br>10-19y: 47.0<br>20-54y: 163.0<br>≥55y: 95.5 | Incidence rate ratio of all-cause AGE inpatient admissions | **5-9y**<br>Years 1-5: 0.64 (0.54-0.75) (p<0.001)<br><br>**10-19y**<br>Years 1-5: 0.57 (0.48-0.68) (p<0.001)<br><br>**20-54y**<br>Years 1-5: 0.80 (0.73-0.89) (p<0.001)<br><br>**≥55y**<br>Years 1-5: 1.17 (1.01-1.35) (p=0.017) |
| [7] | Finland | 2009 | RV5 RotaTeq<br><br><1y^<br>2014: 92%<br>2015: 91%<br>2016: 84%<br>2017: 80% | Retrospective register-based study | Pre-vaccine:<br>2002-2005<br><br>Post-vaccine (Years 5-8):<br>2014-2017 | Inpatient and outpatient children due to AGE in a national healthcare register | 0.5-2y | **Pre-vaccine**<br>6m: 55,376 person-years<br>12m: 55,376 person-years<br>18m: 55,376 person-years<br><br>**Unvaccinated post-vaccine**<br>6m: 4304 person-years<br>12m: 4304 person-years<br>18m: 4304 person-years | Incidence of all-cause AGE inpatient admissions per 1000 person-years<br><br>Indirect vaccine effectiveness against all-cause AGE inpatient admissions | **Incidence** (Years 5-8)<br>6m: 29.27 (pre); 10.92 (post)<br>12m: 20.95 (pre); 8.60 (post)<br>18m: 14.32 (pre); 8.60 (post)<br><br>**Incidence rate ratio** (Years 5-8)<br>6m: 0.37*<br>12m: 0.41*<br>18m: 0.60*<br><br>**Indirect vaccine effectiveness** (Years 5-8)<br>56.4% (55.6-57.3) |
| [9] | Ghana | Apr 2012 | RV1 Rotarix<br><br>2 doses | Hospital-based surveillance | Pre-vaccine:<br>Jan 2009 - Mar 2012 | Children admitted to two teaching hospitals with AGE | <5y | **Pre-vaccine**<br>1y: 386/820 (47%)<br>2-4y: 81/252 (32%) | Incidence rate reduction in all-cause AGE | **1y**<br>Year 0: 30% (0-51) |

| Reference | Country | Date of universal rotavirus vaccine introduction | Vaccine formulation and coverage | Study design | Study period | Study population | Age groups | Case numbers | Outcome and effect measure | Results (95% CI) |
|---|---|---|---|---|---|---|---|---|---|---|
| | | | 2012: 90%<br>2013-14: 93-100% | | Post-vaccine (Years 0-2):<br>Apr 2012 - Dec 2014 | | | **Unvaccinated post-vaccine**<br><br>**1y:**<br>Year 0 = 50/186 (27%)<br>Year 1 = 51/151 (34%)<br><br>**2-4y:**<br>Year 0 = 11/46 (24%)<br>Year 1 = 17/49 (35%) | inpatient admissions | **2-4y**<br>Year 0: 30% (-18 to 60)<br>Year 1: 9% (-123 to 63) |
| 30 | Kiribati | Aug 2015 | RV1 Rotarix<br><br>2 doses<br>2016: 79%<br>2017: 91% | Retrospective review | Pre-vaccine:<br>Jan 2010 - Dec 2013<br><br>Post-vaccine (Years 1-2):<br>Jan 2016 - Sep 2017 | Children admitted to two hospitals with AGE or outpatient presentations from hospitals and clinics nationwide | <5y | **Pre-vaccine**<br>431/3361 (12.8%)<br><br>**Unvaccinated post-vaccine**<br>102/1407 (7.2%) | Incidence rate of all-cause AGE inpatient admissions per 100,000 | **Incidence rate**<br><br>**Pre-vaccine**<br>2-4y: 299 (232-379)<br><br>**Post-vaccine**<br>2-4y: 261 (174-378)<br><br>**Incidence rate ratio**<br><br>**2-4y**<br>Years 1-2: 0.87* |
| 31 | Norway | Oct 2014 | RV1 Rotarix<br><br>2 doses<br>Year 1: 82% | Laboratory-based study | Pre-vaccine:<br>Jul 2009 - Jun 2015<br><br>Post-vaccine (Years 0-3):<br>Jul 2015 - Jun 2018 | Children admitted to five hospitals with AGE | <5y | **Pre-vaccine**<br>20,786/1,852,177 person-years (total in all age groups)<br><br>**Unvaccinated post-vaccine**<br>5007/912,977 person-years (total in all age groups) | Incidence rate ratio of all-cause AGE inpatient admissions | **2y**<br>Year 0: 0.47 (0.40-0.56; p<0.05)<br><br>**3y**<br>Year 0: 0.53 (0.43-0.67; p<0.05)<br>Year 1: 0.70 (0.56-0.87; p<0.05)<br><br>**4y**<br>Year 0: 0.61 (0.48-0.78; p<0.05)<br>Year 1: 0.65 (0.50-0.83; p<0.05)<br>Year 2: 0.70 (0.53-0.92; p<0.05) |
| 32 | Senegal | Nov 2014 | RV1 Rotarix<br><br>2015: 89%<br>2016: 83% | Hospital-based surveillance | Pre-vaccine:<br>Mar 2010 - Feb 2014<br><br>Post-vaccine (Years 0-1):<br>Mar 2015 - Feb 2017 | Children admitted to a tertiary hospital with AGE | <5y | **Pre-vaccine**<br>1y: ~398/2182<br>2-4y: ~232/3020<br><br>**Unvaccinated post-vaccine**<br>1y: 85/534<br>2-4y: 100/1540 | Percent reduction in all-cause AGE inpatient admissions | **1y**<br>Year 0: 12.7% (p=0.29)<br><br>**2-4y**<br>Year 0: 12.6% (p=0.45)<br>Year 1: 19.1% (p=0.28) |
| 13 | Tanzania | Jan 2013 | RV1 Rotarix<br><br><1y<br>2013: 18% (Apr), 65% (Oct)<br>2014: 85% (Apr), 83% (Oct) | Hospital-based surveillance | Pre-vaccine:<br>2010 - 2012<br><br>Post-vaccine (Years 0-2):<br>2013 - 2015 | Children admitted to a hospital with diarrhoea as the main reason for admission | <5y | **Pre-vaccine**<br>1y: 342<br>2-4y: 189<br><br>**Unvaccinated post-vaccine**<br><br>**1y:**<br>Year 0 = 322<br><br>**2-4y:** | Percent reduction in all-cause AGE inpatient admissions | **1y**<br>Year 0: 6%<br><br>**2-4y**<br>Year 0: 15%<br>Year 1: <1% |

| Reference | Country | Date of universal rotavirus vaccine introduction | Vaccine formulation and coverage | Study design | Study period | Study population | Age groups | Case numbers | Outcome and effect measure | Results (95% CI) |
|---|---|---|---|---|---|---|---|---|---|---|
| | | | | | | | | Year 0 = 160<br>Year 1 = 188 | | |
| [33] | Togo | Jun 2014 | RV1 Rotarix<br><br>2015: 85%<br>2016: 92% | Hospital-based surveillance and retrospective hospital-based review | Pre-vaccine:<br>Jul 2010 – Jun 2014<br><br>Post-vaccine (Years 0-1):<br>Jul 2014 – Jun 2016 | Children examined for AGE at emergency departments of two hospitals and children with all-cause hospitalisations and all-cause AGE hospitalisations identified from paediatric ward registers at five referral and regional hospitals | <5y | **Pre-vaccine**<br>1-4y: 1639/13,216<br><br>**Unvaccinated post-vaccine**<br>1-4y: 304/3254 | Percent reduction in proportion of all-cause AGE inpatient admissions | **1-4y**<br>Year 0: 30.1% (p<0.02) |
| [18] | United States | RV5: Feb 2006<br><br>RV1: Apr 2008 | RV5 RotaTeq and RV1 Rotarix<br><br>≥1 dose<br>2007: 51%<br>2008: 66%<br>2009: 80%<br>2010: 86% | Retrospective register-based study | Pre-vaccine:<br>2001 – 2005<br><br>Post-vaccine (Years 1-4):<br>2007 – 2010 | Children on a commercial health insurance database with at least one outpatient claim# | <20m | **Pre-vaccine**<br>2021/277,893<br><br>**Unvaccinated post-vaccine**<br>Year 1: 271/64,928<br>Year 2: 317/90,882<br>Year 3: 300/61,136<br>Year 4: 109/41,888 | Indirect vaccine effectiveness against all-cause AGE inpatient admissions | **<20m**<br>Year 1: -8% (-24 to 6)<br>Year 2: 24% (15-33)<br>Year 3: 9% (-3 to 19)<br>Year 4: 45% (33-54) |
| [20] | United States | RV5: Feb 2006<br><br>RV1: Apr 2008 | RV5 RotaTeq and RV1 Rotarix<br><br><1y with ≥1 dose<br>2007-2008: NBW 64%; LBW 62%; VLBW 47%<br>2008-2009: NBW 72%; LBW 69%; VLBW 52%<br>2009-2010: NBW 76%; LBW 73%; VLBW 58%<br>2010-2011: NBW 78%; LBW 76%; VLBW 63%<br>2011-2012: NBW 82%; LBW 78%; VLBW 64%<br>2012-2013: NBW 86%; LBW 81%; VLBW 66%<br>2013-2014: NBW 88%; LBW 83%; VLBW 67%<br>2014-2015: NBW: 89%; LBW 84%; | Retrospective register-based study | Pre-vaccine:<br>Jul 2001 – Jun 2006<br><br>Post-vaccine (Years 1-8):<br>Jul 2007 – Jun 2015 | Children on a commercial health insurance database# | <5y | **Pre-vaccine and post-vaccine combined**<br>2,256,000 (total in all age groups) | Incidence rate reduction in all-cause AGE inpatient admissions | **2-4y**<br>Year 1: NBW 29% (26-32); LBW 67% (41-81); VLBW 69% (-10 to 91) |

| Reference | Country | Date of universal rotavirus vaccine introduction | Vaccine formulation and coverage | Study design | Study period | Study population | Age groups | Case numbers | Outcome and effect measure | Results (95% CI) |
|---|---|---|---|---|---|---|---|---|---|---|
| | | | VLBW 68% | | | | | | | |
| [22] | United States | RV5: Feb 2006<br><br>RV1: Apr 2008 | RV5 RotaTeq and RV1 Rotarix<br><br><1y with ≥1 dose<br>2007: 64%<br>2008: 73%<br>2009: 76%<br>2010: 78% | Retrospective register-based study | Pre-vaccine:<br>Jul 2001 – Jun 2006<br><br>Post-vaccine (Years 1-4):<br>Jul 2007 – Jun 2011 | Children on a commercial health insurance database with diarrhoea-associated healthcare events[#] | <5y | **Pre-vaccine and post-vaccine combined**<br>406,000 (total in all age groups) | Incidence rate reduction in all-cause AGE inpatient admissions | **2-4y**<br>Year 1: 41% (38-44) |
| **Outpatient attendances for all-cause AGE** | | | | | | | | | | |
| [5] | Canada | Aug 2011<br><br>RV5 and RV1 in private market from 2006/2007 | RV1 Rotarix and RV5 RotaTeq<br><br><1y^<br>2017: 79% | Retrospective population-based cohort | Pre-vaccine:<br>Aug 2005 – Jul 2011<br><br>Post-vaccine (Years 0-4):<br>Aug 2011 – Mar 2016 | Ontarians with a valid health card that utilised healthcare services for AGE | All ages | **Pre-vaccine and unvaccinated post-vaccine combined**<br>2779/183,170 (total in all age groups) | Incidence rate ratio of all-cause AGE emergency department outpatient attendances | **5-19y**<br>Years 0-4: 0.90 (0.89-0.92)<br><br>**20-44y**<br>Years 0-4: 1.00 (0.99-1.02)<br><br>**45-64y**<br>Years 0-4: 1.01 (0.99-1.03)<br><br>**≥65y**<br>Years 0-4: 0.98 (0.96-1.00) |
| [7] | Finland | 2009 | RV5 RotaTeq<br><br><1y^<br>2014: 92%<br>2015: 91%<br>2016: 84%<br>2017: 80% | Retrospective register-based study | Pre-vaccine:<br>2002-2005<br><br>Post-vaccine (Years 5-8):<br>2014-2017 | Inpatient and outpatient children due to AGE in a national healthcare register | 0.5-2y | **Pre-vaccine**<br>6m: 55,376 person-years<br>12m: 55,376 person-years<br>18m: 55,376 person-years<br><br>**Unvaccinated post-vaccine**<br>6m: 4304 person-years<br>12m: 4304 person-years<br>18m: 4304 person-years | Incidence of all-cause AGE outpatient attendances per 1000 person-years<br><br>Indirect vaccine effectiveness against all-cause AGE outpatient attendances | **Incidence** (Years 5-8)<br>6m: 25.81 (pre); 16.96 (post)<br>12m: 20.19 (pre); 17.89 (post)<br>18m: 12.98 (pre); 17.89 (post)<br><br>**Incidence rate ratio** (Years 5-8)<br>6m: 0.66*<br>12m: 0.89*<br>18m: 1.38*<br><br>**Indirect vaccine effectiveness**<br>10.6% (9.6-11.6) |
| [22] | United States | RV5: Feb 2006<br><br>RV1: Apr 2008 | RV5 RotaTeq and RV1 Rotarix<br><br><1y with ≥1 dose<br>2007: 64%<br>2008: 73%<br>2009: 76%<br>2010: 78% | Retrospective register-based study | Pre-vaccine:<br>Jul 2001 – Jun 2006<br><br>Post-vaccine (Years 1-4):<br>Jul 2007 – Jun 2011 | Children on a commercial health insurance database with diarrhoea-associated healthcare events[#] | <5y | **Pre-vaccine and post-vaccine combined**<br>406,000 (total in all age groups) | Incidence rate reduction in all-cause AGE outpatient attendances | **2-4y**<br>Year 1: 5% (4-5) |
| **Laboratory-confirmed RV in stool samples** | | | | | | | | | | |
| [34] | Ecuador | Aug 2008 | RV1 Rotarix<br><br>2 doses: 72.1% | Population-based case-control | Pre-vaccine:<br>Jun 2003 - Feb 2008<br><br>Post-vaccine (Years 1-5): | Cases: Community sampling of people with AGE | >5y | **Pre-vaccine**<br>915/23,972 (3.8%)<br><br>**Unvaccinated post-vaccine** | Percent reduction in RV positivity in stool samples | **>5y**<br>Years 1-5: 68.7% (34.1-85.1) |

| Reference | Country | Date of universal rotavirus vaccine introduction | Vaccine formulation and coverage | Study design | Study period | Study population | Age groups | Case numbers | Outcome and effect measure | Results (95% CI) |
|---|---|---|---|---|---|---|---|---|---|---|
| | | | | | Mar 2009 - Jul 2013 | Controls: Household/community members without AGE | | 114/10,831 (1.0%) | | |
| 35 | Eswatini / Swaziland | May 2015 | RV1 Rotarix<br><br><1y^<br>2015: 36%<br>2016: 95% | Hospital-based surveillance | Pre-vaccine:<br>Jan 2013 – Dec 2014<br><br>Transition (Year 0):<br>Jan – Dec 2015<br><br>Post-vaccine (Year 1):<br>Jan – Dec 2016 | Children presenting with diarrhoea as a primary illness and admitted to hospital or treated at the emergency unit in two urban referral hospitals | <5y | **Pre-vaccine**<br>1y: 42/76<br>2-4y: 14/26<br><br>**Unvaccinated post-vaccine**<br><br>**1y**<br>Year 0: 16/42<br><br>**2-4y**<br>Year 0: 1/7<br>Year 1: 3/14 | Percent reduction in RV positivity in stool samples | **1y**<br>Year 0: 31%*<br><br>**2-4y**<br>Year 0: 74%*<br>Year 1: 61%* |
| 36 | Ireland | Dec 2016 | RV1 Rotarix<br><br><1y^<br>2017-2019: 89% | Retrospective observational study | Pre-vaccine:<br>Jan 2015 – Dec 2016<br><br>Post-vaccine (Years 0-2):<br>Jan 2017 – Dec 2019 | Patients with AGE from a national database | ≥65y | **Pre-vaccine**<br>2015: 78/4196 (1.86%)<br>2016: 57/5216 (1.09%)<br><br>**Unvaccinated post-vaccine**<br>2017: 50/4386 (1.14%)<br>2018: 22/4315 (0.51%)<br>2019: 25/4480 (0.56%) | Percent RV positivity in stool samples | **Percent positivity**<br><br>**Pre-vaccine**<br>2015: 1.86%<br>2016: 1.09%<br>Combined: 1.43%<br><br>**Post-vaccine**<br>2017: 1.14%<br>2018: 0.51%<br>2019: 0.56%<br><br>**Incidence rate ratio**<br><br>**≥65y**<br>Year 0: 0.80*<br>Year 1: 0.36*<br>Year 2: 0.39* |
| 37 | Mozambique | Sep 2015 | RV1 Rotarix<br><br>2016: 76%<br>2017: 89% | Hospital-based surveillance | Pre-vaccine:<br>Jan 2014 - Dec 2015<br><br>Post-vaccine (Years 1-2):<br>Jan 2016 - Sep 2017 | Outpatients and inpatients with acute diarrhoea at six hospitals | <5y | **Pre-vaccine**<br><br>**1y**<br>2014: 8/29<br>2015: 75/224<br><br>**2-4y**<br>2014: 1/4<br>2015: 10/64<br><br>**Unvaccinated post-vaccine**<br><br>**1y**<br>2016: 19/145<br><br>**2-4y**<br>2016: 5/71 | Percent RV positivity in stool samples | **Percent positivity**<br><br>**Pre-vaccine**<br><br>**1y**<br>2014: 27.6%<br>2015: 33.5%<br>Combined: 32.8%<br><br>**2-4y**<br>2014: 25.0%<br>2015: 15.6%<br>Combined: 16.2%<br><br>**Post-vaccine**<br><br>**1y** |

| Reference | Country | Date of universal rotavirus vaccine introduction | Vaccine formulation and coverage | Study design | Study period | Study population | Age groups | Case numbers | Outcome and effect measure | Results (95% CI) |
|---|---|---|---|---|---|---|---|---|---|---|
| | | | | | | | | 2017: 1/26 | | 2016: 13.1%<br><br>**2-4y**<br>2016: 7.0%<br>2017: 3.8%<br><br>**Incidence rate ratio**<br><br>**1y**<br>Year 1: 0.40*<br><br>**2-4y**<br>Year 1: 0.43*<br>Year 2: 0.23* |
| 38 | Mozambique | Sep 2015 | RV1 Rotarix<br><br>2016: 46% | Hospital-based surveillance | Pre-vaccine:<br>Mar - Dec 2015<br><br>Post-vaccine (Years 1-4):<br>Jan 2016 - Dec 2019 | Children presenting to a hospital with diarrhoea (inpatients and outpatients) | <5y | **Pre-vaccine**<br>67/192<br><br>**Unvaccinated post-vaccine**<br>21/137 | Percent RV positivity in stool samples | **Percent positivity**<br><br>**Pre-vaccine**<br>34.9% (28.5-41.9)<br><br>**Post-vaccine**<br>15.3% (p=0.002)<br><br>**Incidence rate ratio**<br><br>**<5y**<br>Years 1-4: 0.44* |
| 39 | Rwanda | May 2012 | RV5 RotaTeq<br><br><1y<br>2014: 98% | Laboratory-based study | Pre-vaccine:<br>2010-2012<br><br>Post-vaccine (Year 2):<br>2014 | Not available | <5y | Not available | Percent RV positivity in stool samples | **Percent positivity**<br><br>**Pre-vaccine**<br>34%<br><br>**Post-vaccine**<br>27%<br><br>**Incidence rate ratio**<br><br>**<5y**<br>Year 2: 0.79* |
| 32 | Senegal | Nov 2014 | RV1 Rotarix<br><br>2015: 89%<br>2016: 83% | Hospital-based surveillance | Pre-vaccine:<br>Mar 2010 - Feb 2014<br><br>Post-vaccine (Years 0-1):<br>Mar 2015 - Feb 2017 | Children admitted to a tertiary hospital with AGE | <5y | **Pre-vaccine**<br>1y: ~39/81<br>2-4y: ~15/63<br><br>**Unvaccinated post-vaccine**<br>1y: 5/33<br>2-4y: 5/56 | Percent reduction in RV positivity in stool samples | **1y**<br>Year 0: 68.5% (p=<0.01)<br><br>**2-4y**<br>Year 0: 70% (p=0.12)<br>Year 1: 55% (p=0.25) |
| 40 | Tajikistan | Jan 2015 | RV1 Rotarix<br><br>Complete course | Hospital-based surveillance | Pre-vaccine:<br>Jan 2013 – Dec 2014 | Children admitted for AGE in a paediatric infectious disease | <5y | **Pre-vaccine**<br>70/264 | Percent reduction in RV positivity in | **2-4y**<br>Year 1: 15% (-36 to 48) (p=0.51) |

| Reference | Country | Date of universal rotavirus vaccine introduction | Vaccine formulation and coverage | Study design | Study period | Study population | Age groups | Case numbers | Outcome and effect measure | Results (95% CI) |
|---|---|---|---|---|---|---|---|---|---|---|
| | | | 2016: >95% | | Post-vaccine (Years 1-4):<br>Jan 2016 – Dec 2019 | hospital | | **Unvaccinated post-vaccine**<br>25/115 | stool samples | |
| [23] | United States | RV5: Feb 2006<br><br>RV1: Apr 2008 | RV5 RotaTeq and RV1 Rotarix<br><br><5y (≥1 dose)<br>2008: 32% | Laboratory-based study | Post-vaccine, pre-impact (Years 0-1):<br>Feb 2006 - May 2007<br><br>Post-vaccine impact (Years 2-4):<br>Feb 2008 - May 2010 | Adults with stool samples collected for bacterial stool culture, acting as a surrogate for clinically significant diarrhoea | ≥18y | **Pre-vaccine**<br>43/989 (4.35%)<br><br>**Unvaccinated post-vaccine**<br>57/2541 (2.24%) | Percent reduction in RV positivity in stool samples | **≥18y**<br><br>Overall<br>Years 2-4: 48.4% (p=0.0007)<br><br>Inpatient admissions only<br>Years 2-4: 42.5% (p=0.042)<br><br>Outpatient attendances only<br>Years 2-4: 49.3% (p=0.016) |
| **Mixed outcomes** | | | | | | | | | | |
| [34] | Ecuador | Aug 2008 | RV1 Rotarix<br><br>2 doses: 72.1% | Population-based case-control | Pre-vaccine:<br>Jun 2003 - Feb 2008<br><br>Post-vaccine (Years 1-5):<br>Mar 2009 - Jul 2013 | Cases: Community sampling of people with AGE<br><br>Controls: Household/community members without AGE | >5y | **Pre-vaccine**<br>915/23,972 (3.8%)<br><br>**Unvaccinated post-vaccine**<br>114/10,831 (1.0%) | Percent reduction in all-cause AGE in the community | **>5y**<br>Years 1-5: 17.4% (-16.9 to 41.6) |
| [41,42] | Finland | Sep 2009 | RV5 RotaTeq<br><br>2009-2011: 95-97%<br>2012-2014: 86% | Hospital-based surveillance | Pre-vaccine:<br>Sep 2006 - Aug 2008<br><br>Post-vaccine:<br>Sep 2009 - Aug 2011 (Years 0-2);<br>Sep 2012 - Aug 2014 (Years 3-5) | Children with AGE presenting to emergency or admitted to a hospital (combination of inpatient admissions and emergency outpatient attendances) | <16y | **Pre-vaccine**<br>243<br><br>**Unvaccinated post-vaccine**<br>2009-2011: 70<br>2012-2014: 15 | Percent reduction in RV-AGE inpatient admissions and emergency outpatient attendances | **Children too old to be vaccinated**<br>Years 0-2: 72%<br>Years 3-5: 94% (p<0.001) |
| [43] | Israel | Jan 2011<br><br>RV1 and RV5 in private market from 2007 | RV5 RotaTeq<br><br>≥1 dose<br>2012: Jewish 95%; Bedouin 90% | Prospective population- and hospital-based observational study | Pre-vaccine:<br>Apr 2006 – Mar 2008<br><br>Limited vaccine:<br>Apr 2008 – Mar 2011<br><br>Post-vaccine (Years 0-1):<br>Apr 2011 – Mar 2013 | Children with history of diarrhoea or vomiting presenting to a paediatric emergency room | <5y | **Pre-vaccine and unvaccinated post-vaccine combined**<br>2340/150,229 (total in all age groups) | Incidence rate reduction of RV-AGE inpatient admissions and outpatient attendances<br><br>Incidence rate reduction of all-cause AGE inpatient admissions and outpatient attendances | **RV-AGE**<br><br>**1y**<br>Year 0: Jewish 70% (58-78); Bedouin 21%<br><br>**2-4y**<br>Year 0: Jewish 36% (8-62); Bedouin 15%<br>Year 1: Jewish 46% (7-69); Bedouin -14%<br><br>**All-cause AGE**<br><br>**1y**<br>Year 0: Jewish 26% (18-33); Bedouin 30%<br><br>**2-4y**<br>Year 0: Jewish 11% (1-21); |

| Reference | Country | Date of universal rotavirus vaccine introduction | Vaccine formulation and coverage | Study design | Study period | Study population | Age groups | Case numbers | Outcome and effect measure | Results (95% CI) |
|---|---|---|---|---|---|---|---|---|---|---|
| | | | | | | | | | | Bedouin 25%<br>Year 1: Jewish 30% (17-41); Bedouin 4% |
| [44] | Malawi | Oct 2012 | RV1 Rotarix<br><br>2016: 99-100% | Hospital-based surveillance | Pre-vaccine:<br>Dec 2011 – Oct 2012<br><br>Post-vaccine (Years 0-6):<br>Nov 2012 – Oct 2019 | Children presenting with AGE to a referral hospital | <5y | **RV-AGE**<br><br>**Pre-vaccine**<br>176<br><br>**Unvaccinated post-vaccine**<br>177<br><br>**All-cause AGE**<br><br>**Pre-vaccine**<br>401<br><br>**Unvaccinated post-vaccine**<br>534 | Percent reduction in RV-AGE inpatient admissions and outpatient attendances<br><br>Percent reduction in all-cause AGE inpatient admissions and outpatient attendances | **RV-AGE**<br><br>**<6m**<br>Years 0-6: 47%* (p=0.360)<br><br>**6-11m**<br>Years 0-6: 34%* (p=0.352)<br><br>**1y**<br>Years 0-6: -120%* (p=0.364)<br><br>**2-4y**<br>Years 0-6: -700%* (p=0.496)<br><br>**All-cause AGE**<br><br>**<6m**<br>Years 0-6: 32%* (p=0.488)<br><br>**6-11m**<br>Years 0-6: 10%* (p=0.228)<br><br>**1y**<br>Years 0-6: -131%* (p=0.190)<br><br>**2-4y**<br>Years 0-6: -166%* (p=0.084) |

**Calculated from raw values available. Percent reduction = percent in post-vaccine period – percent in pre-vaccine period. Incidence rate ratio = incidence or percent positivity in post-vaccine period / incidence or percent positivity in pre-vaccine period.*

*^Vaccine coverage data was obtained from the World Health Organization Global Health Observatory (https://apps.who.int/gho/data/node.main.ROTACn) if they were not reported in the paper.*

*[#]These studies used the same data source but performed different analyses*

**Supplementary S5. Risk of bias assessment of included studies using the ROBINS-E tool.**

| Author and year | Bias due to confounding | Bias due to measurement of the exposure | Bias in selection of participants into the study | Bias due to post-exposure intervention | Bias due to missing data | Bias due to measurement of outcomes | Bias in selection of the reported result | Overall bias |
|---|---|---|---|---|---|---|---|---|
| Abeid 2017[13] | - | + | + | + | + | + | + | - |
| Anderson 2013[23] | X | + | + | + | + | + | + | X |
| Andersson 2016[39] * | | | | | | | | ! |
| Armah 2016[9] | - | + | + | + | + | + | + | - |
| Bennett 2018[26] | - | + | + | + | + | + | + | - |
| Bruun 2021[31] | - | + | + | + | + | + | + | - |
| Chissaque 2021[38] | X | + | + | + | + | + | + | X |
| Dahl 2018[20] | - | + | + | + | + | X | + | - |
| David 2014[2] | - | + | + | + | + | + | X | X |
| de Deus 2018[37] | - | + | + | + | + | + | + | - |
| Diop 2018[32] | - | + | + | + | + | + | + | - |
| Eberly 2011[21] | - | + | + | + | + | X | + | - |
| Enweronu-Laryea 2018[10] | - | + | + | + | + | + | + | - |
| Forrest 2017[16] | - | + | + | + | + | + | + | - |
| Gheorghita 2016[12] | - | + | + | + | + | + | + | - |

| Author and year | Bias due to confounding | Bias due to measurement of the exposure | Bias in selection of participants into the study | Bias due to post-exposure intervention | Bias due to missing data | Bias due to measurement of outcomes | Bias in selection of the reported result | Overall bias |
|---|---|---|---|---|---|---|---|---|
| Givon-Lavi 2015[43] | - | + | + | + | + | + | + | - |
| Hemming 2013[41] | X | + | + | + | + | + | + | X |
| Hemming-Harlo 2016[42] | X | + | + | + | + | + | + | X |
| Hungerford 2016[17] | - | + | + | + | + | + | + | - |
| Jani 2018[14] | - | + | + | + | + | + | + | - |
| Jenney 2021[29] | - | + | + | + | + | + | + | - |
| Koivumagi 2020[6] | - | + | + | + | + | + | X | X |
| Kraay 2020[34] | - | + | + | + | - | + | + | - |
| Lai 2020[30] | - | + | + | + | + | + | + | - |
| Leshem 2014[22] | - | + | + | + | + | + | + | - |
| Mandolo 2021[44] | - | + | + | + | + | + | + | - |
| Maphalala 2018[35] | - | + | + | + | + | + | + | - |
| Marlow 2015[27] | - | + | + | + | X | + | X | X |
| Mpabalwani 2016[24] | - | + | + | + | + | + | + | - |
| Mpabalwani 2018[25] | - | + | + | + | + | + | + | - |
| Mujuru 2017[28] | - | + | + | + | + | + | + | - |

| Author and year | Bias due to confounding | Bias due to measurement of the exposure | Bias in selection of participants into the study | Bias due to post-exposure intervention | Bias due to missing data | Bias due to measurement of outcomes | Bias in selection of the reported result | Overall bias |
|---|---|---|---|---|---|---|---|---|
| Nazurdinov 2022[40] | - | + | + | + | + | + | + | - |
| Panozzo 2014[18] | - | + | + | + | + | + | + | - |
| Paulke-Korinek 2013[3] | - | + | + | + | + | + | + | - |
| Payne 2011[19] | - | + | + | + | + | + | + | - |
| Pietsch 2019[8] | - | + | + | + | - | + | + | - |
| Safadi 2010[4] | - | + | + | + | - | + | + | - |
| Sahakyan 2016[1] | - | + | + | + | + | + | + | - |
| Solastie 2020[7] | - | + | + | + | X | + | + | X |
| Tharmaphornpilas 2017[15] | - | + | + | + | + | + | + | - |
| Tsolenyanu 2018[33] | - | + | + | + | + | + | + | - |
| Wandera 2017[11] | - | + | + | + | + | + | + | - |
| Wilson 2019[5] | - | + | + | + | + | + | + | - |
| Yandle 2021[36] | - | + | + | + | + | + | + | - |

+ Low risk of bias    - Some concerns    X High risk of bias    ! Very high risk of bias

**In line with ROBINS-E instructions, studies with an overall very high risk of bias were assessed as such during preliminary consideration. If authors did not attempt to control for confounding and there was sufficient potential confounding that an unadjusted result should not be considered further, the study was classified as having a very high risk of bias and individual domains of bias were not assessed.*

**Supplementary S6. Subgroup analyses of distribution of the indirect effects of universal infant rotavirus vaccine introduction.**

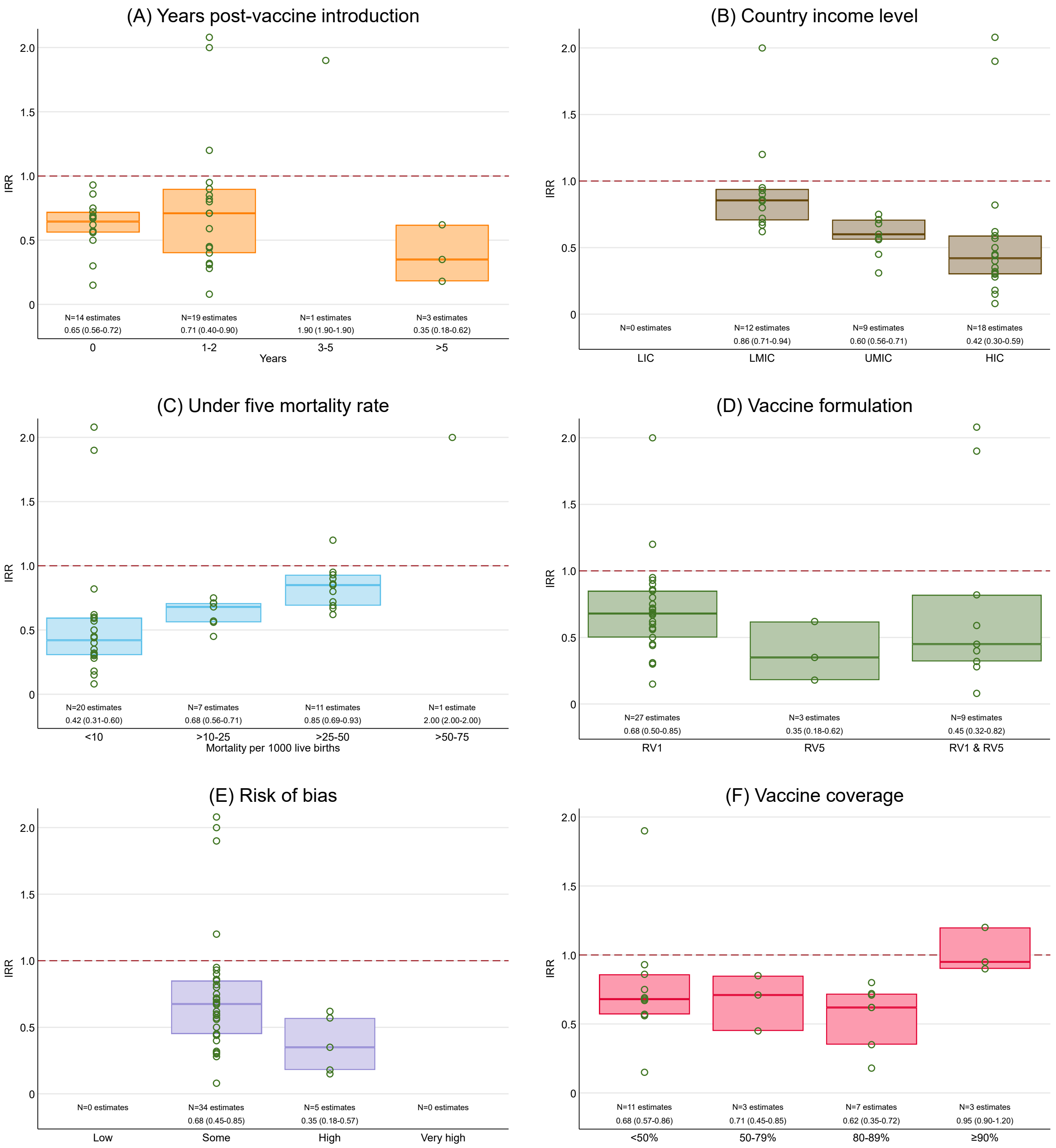


**Supplementary S6 Figure S1. Distribution of the indirect effects of universal infant rotavirus vaccine introduction on laboratory-confirmed rotavirus acute gastroenteritis (RV-AGE) inpatient admissions in unvaccinated children <5 years by (A) years post-vaccine introduction, (B) country income level, (C) national under-five mortality rate, (D) vaccine formulation, (E) study risk of bias classification, and (F) vaccine coverage.** The effect measure is incidence rate ratios (IRR) of RV-AGE inpatient admissions between the post- vs pre-vaccine introduction periods. The boxes indicate the medians (centre line) and interquartile ranges (top and bottom lines) for each category. The number of individual estimates and median (interquartile range) for each category are shown. LIC (low), LMIC (lower-middle), UMIC (upper-middle), HIC (high).

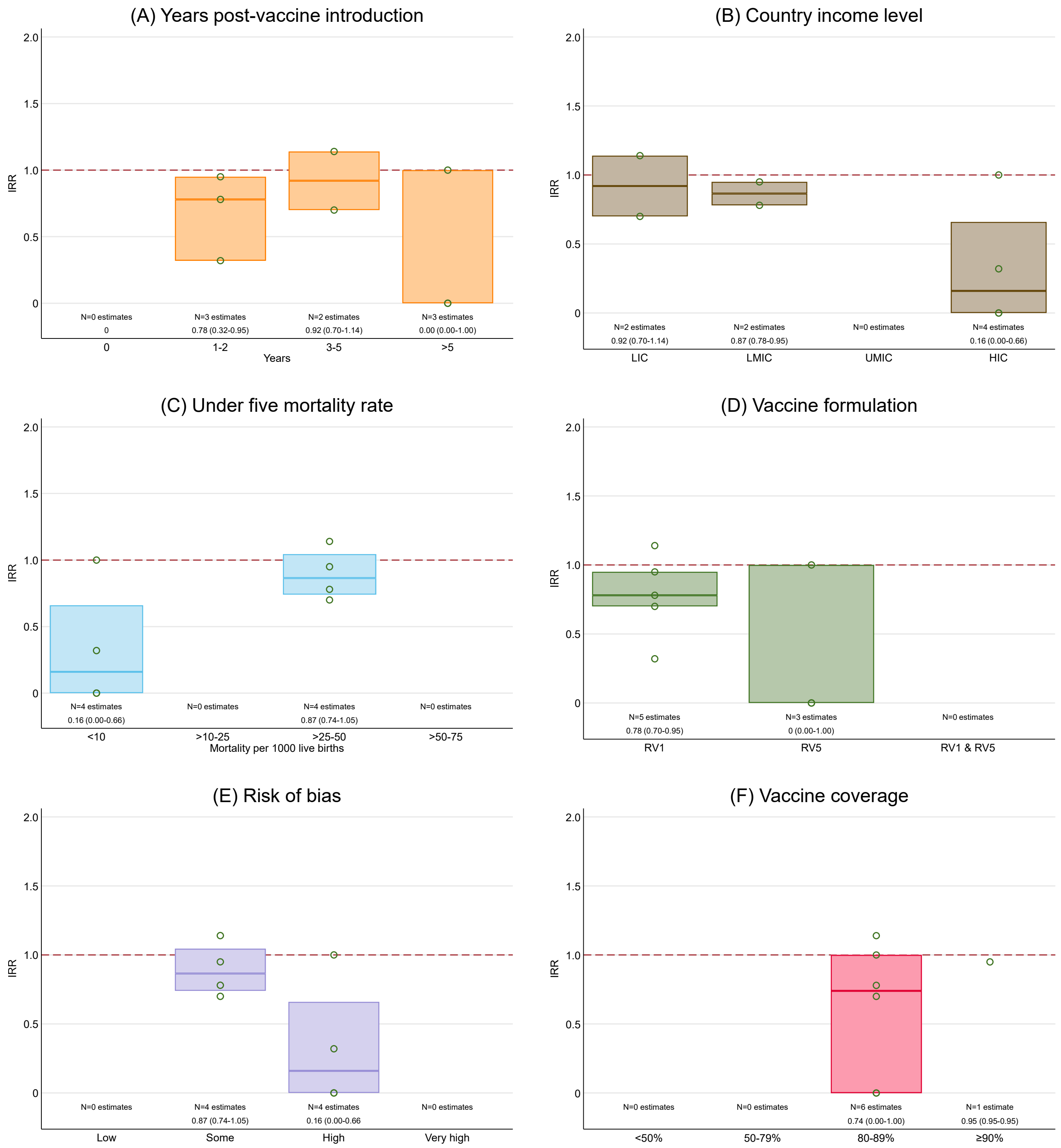


**Supplementary S6 Figure S2. Distribution of the indirect effects of universal infant rotavirus vaccine introduction on laboratory-confirmed rotavirus acute gastroenteritis (RV-AGE) outpatient attendances in unvaccinated children <5 years by (A) years post-vaccine introduction, (B) country income level, (C) national under-five mortality rate, (D) vaccine formulation, (E) study risk of bias classification, and (F) vaccine coverage.** The effect measure is incidence rate ratios (IRR) of RV-AGE outpatient attendances between the post- vs pre-vaccine introduction periods. The boxes indicate the medians (centre line) and interquartile ranges (top and bottom lines) for each category. The number of individual estimates and median (interquartile range) for each category are shown. LIC (low), LMIC (lower-middle), UMIC (upper-middle), HIC (high).

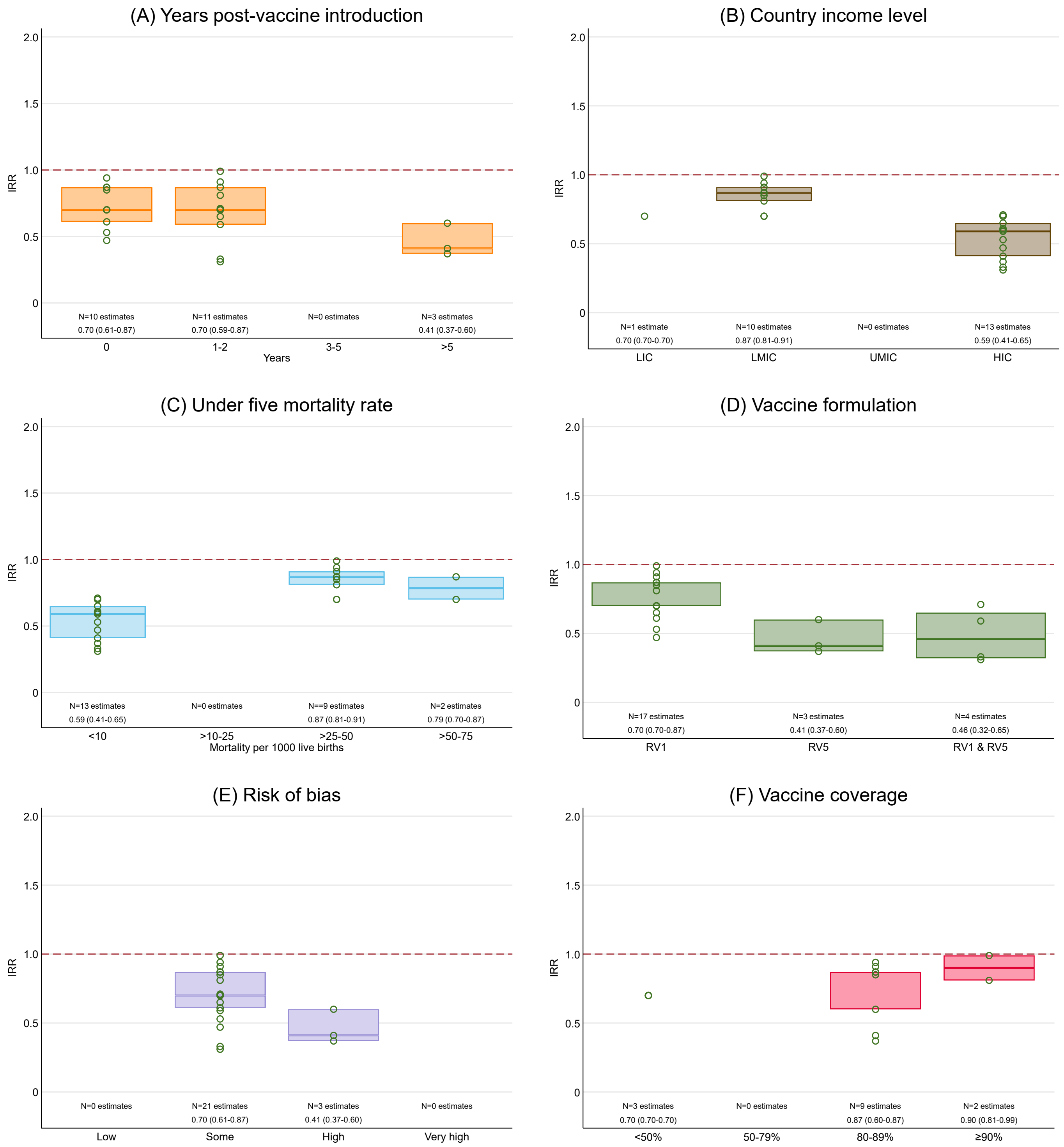


**Supplementary S6 Figure S3. Distribution of the indirect effects of universal infant rotavirus vaccine introduction on all-cause acute gastroenteritis (AGE) inpatient admissions in unvaccinated children <5 years by (A) years post-vaccine introduction, (B) country income level, (C) national under-five mortality rate, (D) vaccine formulation, (E) study risk of bias classification, and (F) vaccine coverage.** The effect measure is incidence rate ratios (IRR) of all-cause AGE inpatient admissions between the post- vs pre-vaccine introduction periods. The boxes indicate the medians (centre line) and interquartile ranges (top and bottom lines) for each category. The number of individual estimates and median (interquartile range) for each category are shown. LIC (low), LMIC (lower-middle), UMIC (upper-middle), HIC (high).

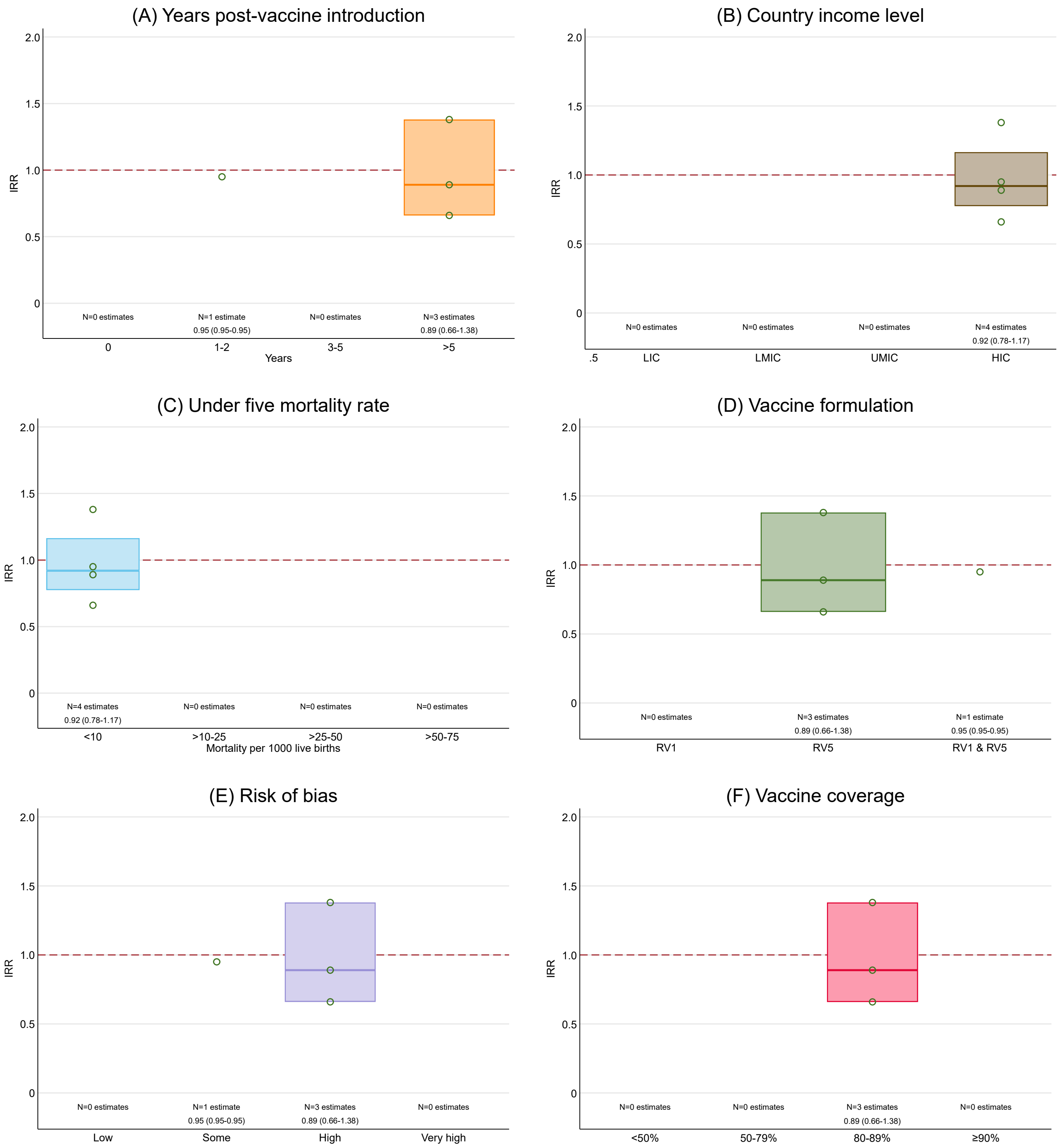


**Supplementary S6 Figure S4. Distribution of the indirect effects of universal infant rotavirus vaccine introduction on all-cause acute gastroenteritis (AGE) outpatient attendances in unvaccinated children <5 years by (A) years post-vaccine introduction, (B) country income level, (C) national under-five mortality rate, (D) vaccine formulation, (E) study risk of bias classification, and (F) vaccine coverage.** The effect measure is incidence rate ratios (IRR) of all-cause AGE outpatient attendances between the post- vs pre-vaccine introduction periods. The boxes indicate the medians (centre line) and interquartile ranges (top and bottom lines) for each category. The number of individual estimates and median (interquartile range) for each category are shown. LIC (low), LMIC (lower-middle), UMIC (upper-middle), HIC (high).

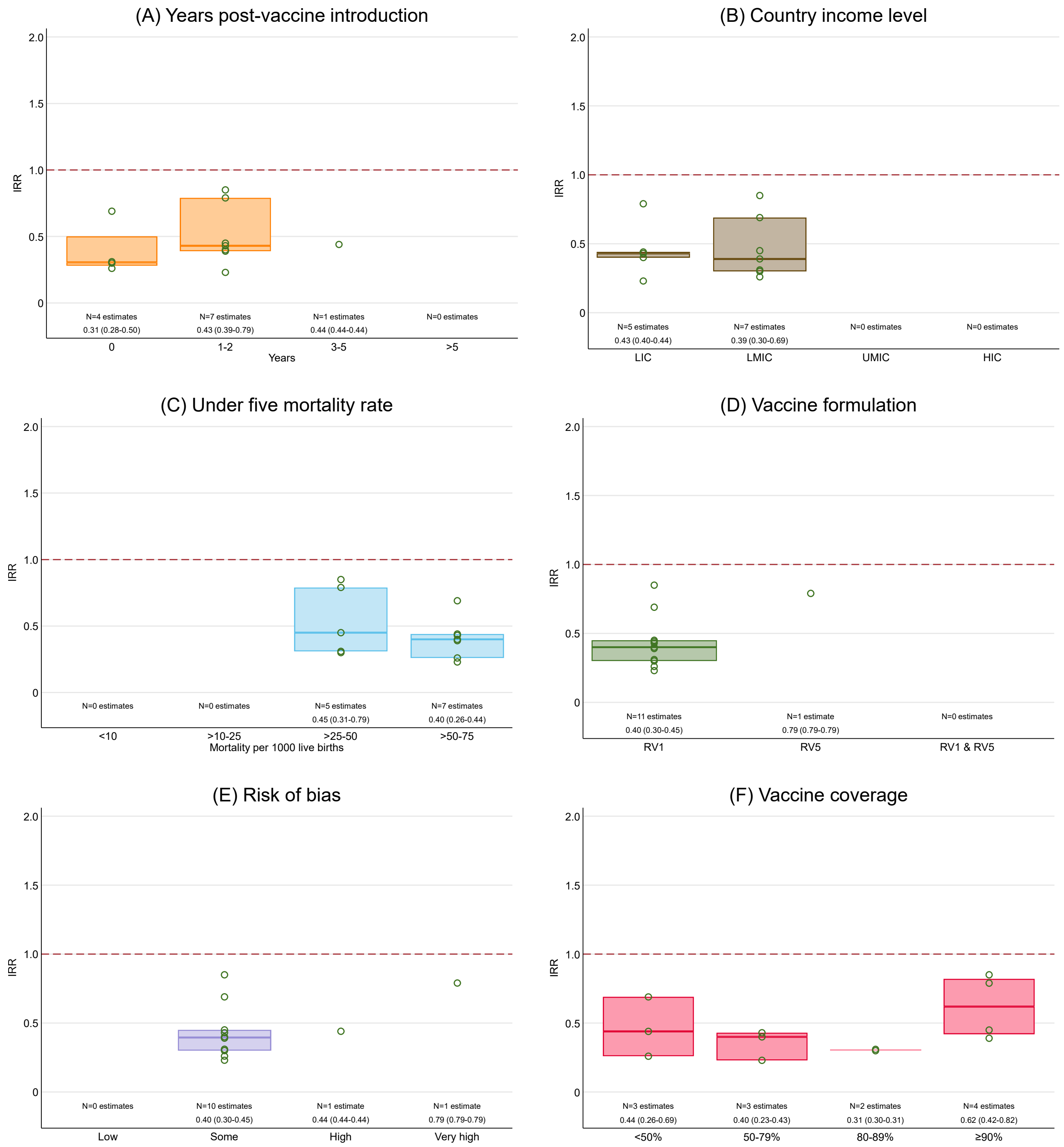


**Supplementary S6 Figure S5. Distribution of the indirect effects of universal infant rotavirus vaccine introduction on laboratory-confirmed rotavirus positivity in stool samples in unvaccinated children <5 years by (A) years post-vaccine introduction, (B) country income level, (C) national under-five mortality rate, (D) vaccine formulation, (E) study risk of bias classification, and (F) vaccine coverage.** The effect measure is incidence rate ratios (IRR) of stool rotavirus positivity between the post- vs pre-vaccine introduction periods. The boxes indicate the medians (centre line) and interquartile ranges (top and bottom lines) for each category. The number of individual estimates and median (interquartile range) for each category are shown. LIC (low), LMIC (lower-middle), UMIC (upper-middle), HIC (high).

**Supplementary S7. Sensitivity analysis of distribution of the indirect effects of universal infant rotavirus vaccine introduction, excluding data points that only reported data from zero years post-introduction.**

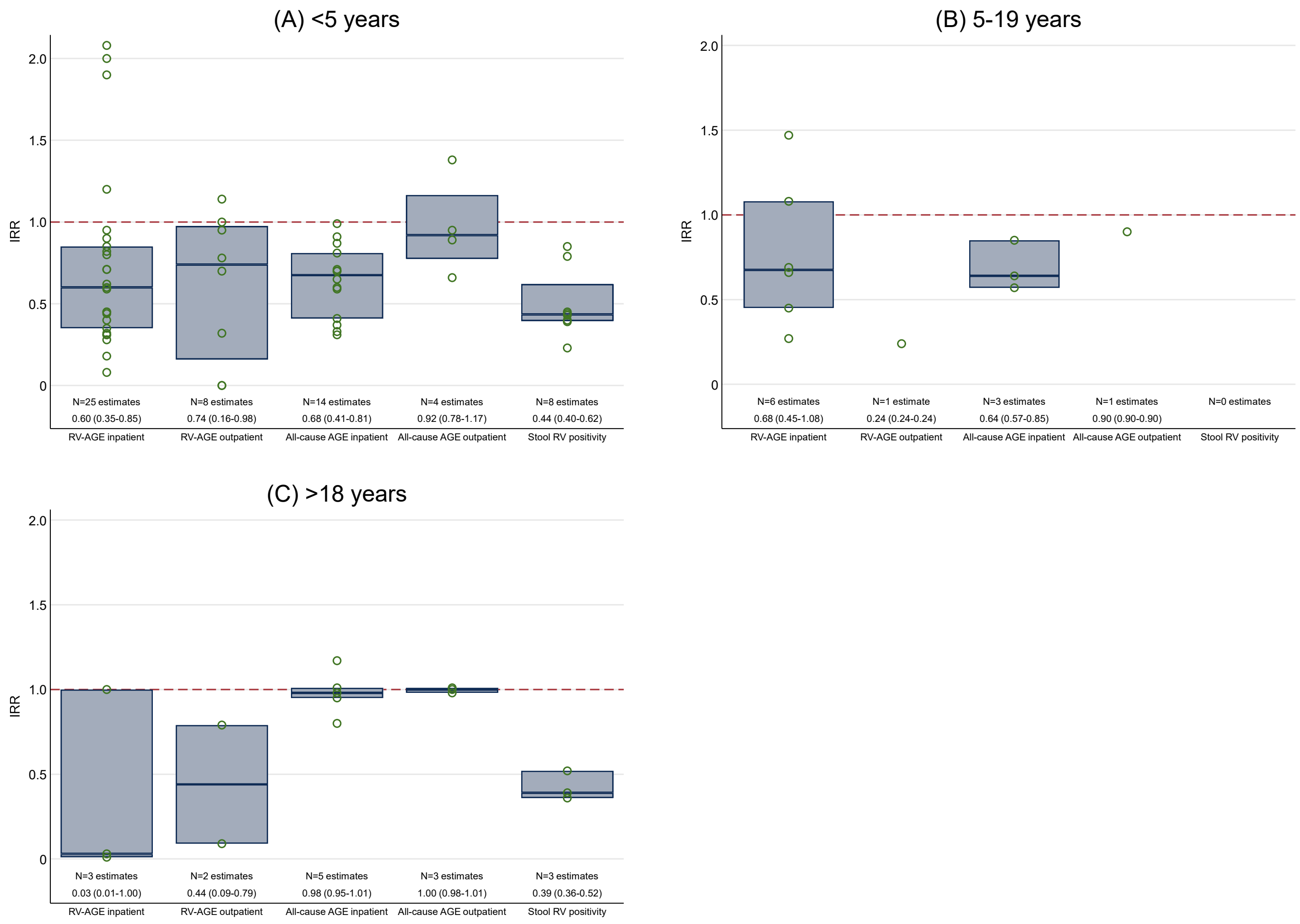


**Supplementary S7 Figure S6. Distribution of the indirect effects of universal infant rotavirus vaccine introduction, excluding data points that only reported data from zero years post-introduction, in (A) unvaccinated children <5 years, (B) children and adolescents 5-19 years, and (C) adults >18 years.** Outcome measures shown are incidence rate ratios (IRR) of laboratory-confirmed rotavirus-specific acute gastroenteritis (RV-AGE) inpatient admissions, laboratory-confirmed RV-AGE outpatient attendances, all-cause AGE inpatient admissions, all-cause AGE outpatient attendances, and laboratory-confirmed RV positivity in stool samples, between the post- vs pre-vaccine introduction periods. The boxes indicate the medians (centre line) and interquartile ranges (top and bottom lines) for each category. The number of individual estimates and median (interquartile range) for each outcome measure are shown. Number of studies reporting the outcome: RV-AGE inpatient admissions (n=19), RV-AGE outpatient attendances (n=5), all-cause AGE inpatient admissions (n=10), all-cause AGE outpatient attendances (n=3), stool RV positivity (n=9).

## References


1. Sahakyan G, Grigoryan S, Wasley A, Mosina L, Sargsyan S, Asoyan A, et al. Impact and effectiveness of monovalent rotavirus vaccine in Armenian children. *Clin Infect Dis* 2016; 62 Suppl 2: S147-54.
2. David RL, Kirk MD. Rotavirus gastroenteritis hospitalisations following introduction of vaccination, Canberra. *Communicable Diseases Intelligence Quarterly Report* 2014; 38(1): E3-E8.
3. Paulke-Korinek M, Kollaritsch H, Aberle SW, Zwazl I, Schmidle-Loss B, Vecsei A, et al. Sustained low hospitalization rates after four years of rotavirus mass vaccination in Austria. *Vaccine* 2013; 31(24): 2686-91.
4. Safadi MA, Berezin EN, Munford V, Almeida FJ, de Moraes JC, Pinheiro CF, et al. Hospital-based surveillance to evaluate the impact of rotavirus vaccination in Sao Paulo, Brazil. *Pediatr Infect Dis J* 2010; 29(11): 1019-22.
5. Wilson SE, Rosella LC, Wang J, Renaud A, Le Saux N, Crowcroft NS, et al. Equity and impact: Ontario's infant rotavirus immunization program five years following implementation. A population-based cohort study. *Vaccine* 2019; 37(17): 2408-14.
6. Koivumagi K, Toompere K, Soeorg H, Kallas E, Jogeda EL, Huik K, et al. Acute gastroenteritis hospitalizations after implementation of universal mass vaccination against rotavirus. *Vaccine* 2020; 38(13): 2879-86.
7. Solastie A, Leino T, Ollgren J. Success ofrotavirus vaccination in Finland, a register based study measuring impact beyond overall effectiveness. *Vaccine* 2020; 38(21): 3766-72.
8. Pietsch C, Liebert UG. Rotavirus vaccine effectiveness in preventing hospitalizations due to gastroenteritis: a descriptive epidemiological study from Germany. *Clin Microbiol Infect* 2019; 25(1): 102-6.
9. Armah G, Pringle K, Enweronu-Laryea CC, Ansong D, Mwenda JM, Diamenu SK, et al. Impact and Effectiveness of Monovalent Rotavirus Vaccine Against Severe Rotavirus Diarrhea in Ghana. *Clin Infect Dis* 2016; 62 Suppl 2(Suppl 2): S200-7.
10. Enweronu-Laryea CC, Armah G, Sagoe KW, Ansong D, Addo-Yobo E, Diamenu SK, et al. Sustained impact of rotavirus vaccine introduction on rotavirus gastroenteritis hospitalizations in children <5 years of age, Ghana, 2009-2016. *Vaccine* 2018; 36(47): 7131-4.
11. Wandera EA, Mohammad S, Bundi M, Komoto S, Nyangao J, Kathiiko C, et al. Impact of rotavirus vaccination on rotavirus and all-cause gastroenteritis in peri-urban Kenyan children. *Vaccine* 2017; 35(38): 5217-23.
12. Gheorghita S, Birca L, Donos A, Wasley A, Birca I, Cojocaru R, et al. Impact of Rotavirus Vaccine Introduction and Vaccine Effectiveness in the Republic of Moldova. *Clin Infect Dis* 2016; 62 Suppl 2(Suppl 2): S140-6.
13. Abeid KA, Jani B, Cortese MM, Kamugisha C, Mwenda JM, Pandu AS, et al. Monovalent Rotavirus Vaccine Effectiveness and Impact on Rotavirus Hospitalizations in Zanzibar, Tanzania: Data From the First 3 Years After Introduction. *J Infect Dis* 2017; 215(2): 183-91.
14. Jani B, Hokororo A, McHomvu J, Cortese MM, Kamugisha C, Mujuni D, et al. Detection of rotavirus before and after monovalent rotavirus vaccine introduction and vaccine effectiveness among children in mainland Tanzania. *Vaccine* 2018; 36(47): 7149-56.
15. Tharmaphornpilas P, Jiamsiri S, Boonchaiya S, Rochanathimoke O, Thinyounyong W, Tuntiwitayapun S, et al. Evaluating the first introduction of rotavirus vaccine in Thailand: Moving from evidence to policy. *Vaccine* 2017; 35(5): 796-801.
16. Forrest R, Jones L, Willocks L, Hardie A, Templeton K. Impact of the introduction of rotavirus vaccination on paediatric hospital admissions, Lothian, Scotland: a retrospective observational study. *Arch Dis Child* 2017; 102(4): 323-7.
17. Hungerford D, Read JM, Cooke RP, Vivancos R, Iturriza-Gomara M, Allen DJ, et al. Early impact of rotavirus vaccination in a large paediatric hospital in the UK. *J Hosp Infect* 2016; 93(2): 117-20.
18. Panozzo CA, Becker-Dreps S, Pate V, Weber DJ, Jonsson Funk M, Stürmer T, et al. Direct, indirect, total, and overall effectiveness of the rotavirus vaccines for the prevention of gastroenteritis hospitalizations in privately insured US children, 2007-2010. *Am J Epidemiol* 2014; 179(7): 895-909.
19. Payne DC, Staat MA, Edwards KM, Szilagyi PG, Weinberg GA, Hall CB, et al. Direct and indirect effects of rotavirus vaccination upon childhood hospitalizations in 3 US Counties, 2006-2009. *Clinical Infectious Diseases* 2011; 53(3): 245-53.
20. Dahl RM, Curns AT, Tate JE, Parashar UD. Effect of rotavirus vaccination on acute diarrheal hospitalizations among low and very low birth weight US infants, 2001-2015. *Pediatric Infectious Disease Journal* 2018; 37(8): 817-22.
21. Eberly MD, Gorman GH, Eide MB, Olsen CH, Rajnik M. The effect of rotavirus immunization on rotavirus gastroenteritis hospitalization rates in military dependents. *Vaccine* 2011; 29(4): 650-9.
22. Leshem E, Moritz RE, Curns AT, Zhou F, Tate JE, Lopman BA, et al. Rotavirus vaccines and health care utilization for diarrhea in the United States (2007-2011). *Pediatrics* 2014; 134(1): 15-23.

23. Anderson EJ, Shippee DB, Weinrobe MH, Davila MD, Katz BZ, Reddy S, et al. Indirect protection of adults from rotavirus by pediatric rotavirus vaccination. *Clinical Infectious Diseases* 2013; 56(6): 755-60.
24. Mpabalwani EM, Simwaka CJ, Mwenda JM, Mubanga CP, Monze M, Matapo B, et al. Impact of Rotavirus Vaccination on Diarrheal Hospitalizations in Children Aged <5 Years in Lusaka, Zambia. *Clin Infect Dis* 2016; 62 Suppl 2: S183-7.
25. Mpabalwani EM, Simwaka JC, Mwenda JM, Matapo B, Parashar UD, Tate JE. Sustained impact of rotavirus vaccine on rotavirus hospitalisations in Lusaka, Zambia, 2009-2016. *Vaccine* 2018; 36(47): 7165-9.
26. Bennett A, Pollock L, Jere KC, Pitzer VE, Parashar U, Tate JE, et al. Direct and possible indirect effects of vaccination on rotavirus hospitalisations among children in Malawi four years after programmatic introduction. *Vaccine* 2018; 36(47): 7142-8.
27. Marlow R, Muir P, Vipond B, Lyttle M, Trotter C, Finn A. Assessing the impacts of the first year of rotavirus vaccination in the United Kingdom. *Euro Surveill* 2015; 20(48): 30077.
28. Mujuru HA, Yen C, Nathoo KJ, Gonah NA, Ticklay I, Mukaratirwa A, et al. Reduction in Diarrhea- and Rotavirus-related Healthcare Visits Among Children <5 Years of Age After National Rotavirus Vaccine Introduction in Zimbabwe. *Pediatr Infect Dis J* 2017; 36(10): 995-9.
29. Jenney AWJ, Reyburn R, Ratu FT, Tuivaga E, Nguyen C, Covea S, et al. The impact of the rotavirus vaccine on diarrhoea, five years following national introduction in Fiji. *Lancet Reg Health West Pac* 2021; 6: 100053.
30. Lai J, Nguyen C, Tabwaia B, Nikuata A, Baueri N, Timeon E, et al. Temporal decline in diarrhea episodes and mortality in Kiribati children two years following rotavirus vaccine introduction, despite high malnutrition rates: a retrospective review. *BMC Infect Dis* 2020; 20(1): 207.
31. Bruun T, Salamanca BV, Bekkevold T, Dollner H, Gibory M, Gilje AM, et al. Impact of the Rotavirus Vaccination Program in Norway After Four Years With High Coverage. *Pediatr Infect Dis J* 2021; 40(4): 368-74.
32. Diop A, Thiongane A, Mwenda JM, Aliabadi N, Sonko MA, Diallo A, et al. Impact of rotavirus vaccine on acute gastroenteritis in children under 5 years in Senegal: Experience of sentinel site of the Albert Royer Children's Hospital in Dakar. *Vaccine* 2018; 36(47): 7192-7.
33. Tsolenyanu E, Djadou KE, Fiawoo M, Akolly DAE, Mwenda JM, Leshem E, et al. Evidence of the impact of monovalent rotavirus vaccine on childhood acute gastroenteritis hospitalization in Togo. *Vaccine* 2018; 36(47): 7185-91.
34. Kraay ANM, Ionides EL, Lee GO, Trujillo WFC, Eisenberg JNS. Effect of childhood rotavirus vaccination on community rotavirus prevalence in rural Ecuador, 2008-13. *Int J Epidemiol* 2020; 49(5): 1691-701.
35. Maphalala G, Phungwayo N, Masona G, Lukhele N, Tsegaye G, Dube N, et al. Early impact of rotavirus vaccine in under 5year old children hospitalized due to diarrhea, Swaziland. *Vaccine* 2018; 36(47): 7210-4.
36. Yandle Z, Coughlan S, Dean J, Hare D, De Gascun CF. Indirect impact of rotavirus vaccination on viral causes of acute gastroenteritis in the elderly. *J Clin Virol* 2021; 137: 104780.
37. de Deus N, Chilaule JJ, Cassocera M, Bambo M, Langa JS, Sitoe E, et al. Early impact of rotavirus vaccination in children less than five years of age in Mozambique. *Vaccine* 2018; 36(47): 7205-9.
38. Chissaque A, Bauhofer AFL, Cossa-Moiane I, Sitoe E, Munlela B, Joao ED, et al. Rotavirus A infection in pre- and post-vaccine period: Risk factors, genotypes distribution by vaccination status and age of children in Nampula Province, Northern Mozambique (2015-2019). *PLoS ONE [Electronic Resource]* 2021; 16(8): e0255720.
39. Andersson M, Kabayiza JC, Lindh M. Comparison of rotavirus frequency and genotype distribution in Rwanda before and after vaccine introduction. *Journal of Clinical Virology* 2016; 82(Supplement 1): S63.
40. Nazurdinov A, Azizov Z, Mullojonova M, Sadykova U, Mosina L, Singh S, et al. Impact and effectiveness of monovalent rotavirus vaccine in Tajik children. *Vaccine* 2022; 40(26): 3705-12.
41. Hemming M, Rasanen S, Huhti L, Paloniemi M, Salminen M, Vesikari T. Major reduction of rotavirus, but not norovirus, gastroenteritis in children seen in hospital after the introduction of RotaTeq vaccine into the National Immunization Programme in Finland. *Eur J Pediatr* 2013; 172(6): 739-46.
42. Hemming-Harlo M, Markkula J, Huhti L, Salminen M, Vesikari T. Decrease of Rotavirus Gastroenteritis to a Low Level Without Resurgence for Five Years After Universal RotaTeq Vaccination in Finland. *Pediatr Infect Dis J* 2016; 35(12): 1304-8.
43. Givon-Lavi N, Ben-Shimol S, Cohen R, Greenberg D, Dagan R. Rapid impact of rotavirus vaccine introduction to the National Immunization plan in southern Israel: comparison between 2 distinct populations. *Vaccine* 2015; 33(16): 1934-40.
44. Mandolo JJ, Henrion MYR, Mhango C, Chinyama E, Wachepa R, Kanjerwa O, et al. Reduction in Severity of All-Cause Gastroenteritis Requiring Hospitalisation in Children Vaccinated against Rotavirus in Malawi. *Viruses* 2021; 13(12).
